# Magnetic hierarchical flower-like Fe_3_O_4_@ZIF-67/CuNiMn-LDH catalyst with enhanced redox cycle for Fenton-like degradation of Congo red: optimization and mechanism

**DOI:** 10.1007/s11356-023-27430-2

**Published:** 2023-05-23

**Authors:** Abdelazeem S. Eltaweil, Sara S. Bakr, Eman M. Abd El-Monaem, Gehan M. El-Subruiti

**Affiliations:** Chemistry Department, Faculty of Science, Alexandria University, Alexandria, Egypt

**Keywords:** Fenton-like degradation, Congo red, Redox cycle, Mechanism, LDH, MOF

## Abstract

**Abstract:**

A novel flower-like CuNiMn-LDH was synthesized and modified, to obtain a promising Fenton-like catalyst, Fe_3_O_4_@ZIF-67/CuNiMn-LDH, with a remarkable degradation of Congo red (CR) utilizing H_2_O_2_ oxidant. The structural and morphological characteristics of Fe_3_O_4_@ZIF-67/CuNiMn-LDH were analyzed via FTIR, XRD, XPS, SEM-EDX, and SEM spectroscopy. In addition, the magnetic property and the surface’s charge were defined via VSM and ZP analysis, respectively. Fenton-like experiments were implemented to investigate the aptness conditions for the Fenton-like degradation of CR; pH medium, catalyst dosage, H_2_O_2_ concentration, temperature, and the initial concentration of CR. The catalyst exhibited supreme degradation performance for CR to reach 90.9% within 30 min at pH 5 and 25 °C. Moreover, the Fe_3_O_4_@ZIF-67/CuNiMn-LDH/H_2_O_2_ system revealed considerable activity when tested for different dyes since the degradation efficiencies of CV, MG, MB, MR, MO, and CR were 65.86, 70.76, 72.56, 75.54, 85.99, and 90.9%, respectively. Furthermore, the kinetic study elucidated that the CR degradation by the Fe_3_O_4_@ZIF-67/CuNiMn-LDH/H_2_O_2_ system obeyed pseudo-first-order kinetic model. More importantly, the concrete results deduced the synergistic effect between the catalyst components, producing a continuous redox cycle consisting of five active metal species. Eventually, the quenching test and the mechanism study proposed the predominance of the radical mechanism pathway on the Fenton-like degradation of CR by the Fe_3_O_4_@ZIF-67/CuNiMn-LDH/H_2_O_2_ system.

**Graphical Abstract:**

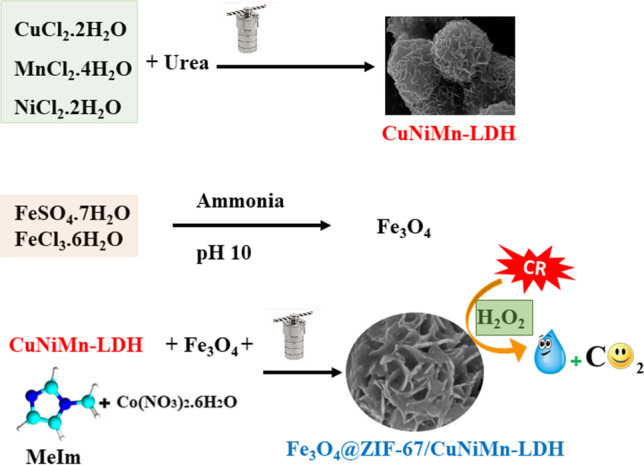

**Supplementary Information:**

The online version contains supplementary material available at 10.1007/s11356-023-27430-2.

## Introduction

The incessant drain of anthropogenic contaminations causes a significant environmental issue especially organic dyes such as methylene blue, reactive orange, reactive yellow, and Congo red that result from diverse industries like leather, textile, the coloring of pharmaceutical drugs, etc. (Eltaweil et al. 2022, Motawea et al. 2022). Dyes have noxious influences on human well-being even with insignificant concentrations, they may lead to teratogenic, allergenic, carcinogenic, and various disorders in human beings, for example, deficiency in the function of the liver, reproductive system, and kidneys (Basha et al. 2022, Dang et al. 2020, Kassem et al. 2021). Notably, azo dyes exhibit around 50% of the overall dyes manufacturing owing to their broad utilization in industries; in addition, they are the bulkiest group of organic dyes in which their structures contain -N=N- group as a chromophore center (Abd El-Monaem et al. 2023, Gomaa et al. 2022, Oladipo et al. 2019).

Congo red (CR) is a type of anion diazo dye and the most periodically utilized one that could metabolize to the carcinogenic benzidine (Dang et al. 2020). The preponderance of CR dye-containing wastewater induced by printing and dyeing industries causes deleterious impacts on the ecological system (sadek Kadari et al. 2022). Thence, it is vital to treat such noxious effluents using an appropriate treatment manner before drainage into the environment (Hussein et al. 2022, Sundararaman et al. 2018). For this sake, versatile approaches have been fostered to overcome the toxic traces of CR dye in wastewater; membrane filtration, adsorption, and advanced oxidation processes (AOPs) (Guo et al. 2020, Ouyang et al. 2019).

AOPs possess special merits compared with other remediation methods, such as simple operation, promising efficiency, and fewer residuum after the treatment. AOPs involve the degradation of the organic residuals via the reactive oxygen species (ROS) such as sulfate radical (SO_4_^•−^) and hydroxyl radical (^•^OH) to nontoxic compounds or the mineralization of them into CO_2_ and H_2_O (Chen et al. 2021). One of the most popular ^•^OH-based AOPs is the Fenton process which has revealed a primer performance in the degradation of several types of persistent dyes like CR (Chu et al. 2020). Such an approach involves the decomposition of hydrogen peroxide (H_2_O_2_) via iron-based catalyst under acidic conditions to produce ^•^OH radicals that could attack the targeted organic dye (Azbar et al. 2004, Lu et al. 2022). Interestingly, the Fenton process is more worthwhile than any other AOP technique, owing to the vastly abundant iron sources and their non-toxicity (Duesterberg &Waite 2006, Gallard et al. 1998). In addition, H_2_O_2_ is a green potent oxidant, since its utilization produces only oxygen and water without giving rise to the formation of secondary pollutants (Su et al. 2020). Nevertheless, the matrix of iron-based materials needs more improvements to boost the generated electron acceptors or positive charge generators, which enhances the Fe^3+^/Fe^2+^ cycle and the generated ^•^OH radicals during the Fenton degradation process (Ouyang et al. 2019).

Layered double hydroxide (LDH) is an anionic clay mineral with superior chemical stability and flexible composition. LDH possesses a special chemical formula, endowing it much interest as a Fenton catalyst; [M^2+^_1-x_ M^3+^_x_(OH)_2_]^x+^(A^n−^_x/n_).mH_2_O, where M^2+^ and M^3+^ are the di and trivalent metal cations, respectively, and A^n−^ expresses the anion in the interlayer (Fan et al. 2021, Sobhana et al. 2017). Interestingly, it was reported that the modification of iron materials by LDH prevents the formation of iron sludge; thereby, LDH could ameliorate the materials’ reusability and the usage of H_2_O_2_ (Wang et al. 2020). A number of LDHs have been prepared from Cu, Ni, and Mn cations; however, there is no study until now involving the fabrication of CuNiMn-LDH. This novel LDH was expected to be a propitious heterogeneous Fenton-like catalyst in which Cu possesses remarkable catalytic activity owing to its low redox potential (Wu et al. 2022b). In addition, Ni could prevent Cu leaching and facilitate the formation of radical species (Khajeh et al. 2022). Furthermore, the multivalence of Ni and Mn atoms displayed astonishing catalytic activity due to the possibility of electron transfer among them (Zhu et al. 2018).

Metal-organic frameworks (MOFs) are ultra-modern materials possessing striking structural characteristics and various features, like outstanding mechanical/thermal stability, adaptable pore size, available unsaturated coordination spots, considerable specific surface area, etc. (Jin et al. 2022, Zhong et al. 2021). Zeolitic imidazolate framework-67 (ZIF-67) is a subcategory of MOFs that acquire a structure analogous to the ancestor inorganic zeolites, consisting of cobalt ion center and imidazolate organic linkers (Wang et al. 2021, Wu et al. 2022a). ZIF-67 features superior chemical/thermal stability, high pores materials, and abundant active sites (Zhang et al. 2021b). Therefore, it has been broadly adapted in various fields, such as separation, adsorption, and catalysis (Wang et al. 2022, Zhang et al. 2022). ZIF-67 has exhibited a promising performance as a Fenton-like catalyst to degrade many organic contaminants. Importantly, several studies have recommended the combination between ZIF-67 and iron-based materials for an effective Fenton-like degradation process owing to the synergistic effect between Fe and Co metals (Chen et al. 2021, Hashemzadeh et al. 2021).

In light of the aforementioned, we aimed to fabricate a novel CuNiMn-LDH and exploit the features of Fe_3_O_4_, and ZIF-67 to enhance its catalytic activity by forming an outstanding heterogeneous Fenton-like catalyst with a continuous redox cycle. It was postulated that the construction of Fe_3_O_4_@ZIF-67/CuNiMn-LDH composite will give rise to a stronger synergistic effect between the contained transition metals, thereby, enhancing the degradation of CR. (i) Fe_3_O_4_@ZIF-67/CuNiMn-LDH and its pure components were characterized by XRD, ZP, XPS, FTIR, VSM, SEM, and SEM-EDX. (ii) A complete Fenton-like study was proceeded to determine the optimum condition to degrade CR by Fe_3_O_4_@ZIF-67/CuNiMn-LDH composite; effect of pH, catalyst dosage, H_2_O_2_ concentration, temperature, and initial concentration of CR. (iii) The catalytic activity of Fe_3_O_4_@ZIF-67/CuNiMn-LDH was evaluated toward different other dyes. (iv) The defining of the radical species and the reaction mechanism were examined by quenching test and XPS of the composite before and after the degradation reaction. (v) The possible degradation pathways of CR by the Fe_3_O_4_@ZIF-67/CuNiMn-LDH/H_2_O_2_ system were identified using GC-MS. (vi) The reusability and metal leaching % of Fe_3_O_4_@ZIF-67/CuNiMn-LDH were examined.

## Experimental section

### Materials

The utilized chemicals are listed in Text S1.

### Synthesis of Fe_3_O_4_

Fe_3_O_4_ nanoparticles were synthesized via a superficial reverse co-precipitation approach (Chen et al. 2018). A total of 3.42 g FeSO_4_.7H_2_O and 3.33 g FeCl_3_.6H_2_O were dissolved in 45 mL distilled water and then transferred into a water bath warmed to 80 °C. For obtaining a homogenous solution, 35.5 μL from concentrated HCl (10 mM) was dipped into the Fe^3+^/Fe^2+^ solution, and then stirred for 1 h. Next, NH_3_.H_2_O was dropped onto the reaction mixture until pH~10 (Eq. 1). The formed solid was left under stirring for 30 min, then it was collected by utilizing a magnet, washed with ultrapure water, and dried at 60 °C for 12 h.

1 $${\mathrm{Fe}}^{2+}+2{\mathrm{Fe}}^{3+}+8\mathrm{O}{\mathrm{H}}^{-}\to {\mathrm{Fe}}_3{\mathrm{O}}_4+4{\mathrm{H}}_2\mathrm{O}$$

### Synthesis of CuNiMn-LDH

CuNiMn-LDH was synthesized via hydrothermal technique; simply, CuCl_2_.2H_2_O, NiCl_2_.2H_2_O, and MnCl_2_.4H_2_O were dissolved in 60 mL of deionized water with a molar ratio of 1:2:1 and 1:3:2 respectively. Next, 10 mmol of urea was added to the above solution under potent stirring for 60 min at room temperature. The cations/urea solution was transferred into an autoclave, sealed carefully, and warmed to 130 °C for 18 h. Finally, the yielded solid was centrifuged, washed with ultrapure water and alcohol, and finally dried at 60 °C for 10 h.

### Synthesis of ZIF-67

A solvothermal approach was applied to prepare ZIF-67 as previously reported by Omer et al. (2021); dissolving Co(NO_3_)_2_.6H_2_O (0.215 g) in 60 mL DMF, then MeIm (0.235 g) was added to the Co^2+^ and retained them under stirring for 1 h. The Co^2+^/MeIm solution was poured into a stainless steel autoclave and put in an oven at 125 °C for 24 h. Ultimately, the outcome mulberry solid was double washed with DMF and ethanol and dried for 12 h at 70 °C.

### Synthesis of Fe_3_O_4_@ZIF-67/CuNiMn-LDH composite

Fe_3_O_4_@ZIF-67/CuNiMn-LDH catalyst was fabricated with different mass ratios between ZIF-67: CuNiMn-LDH; 1:1, 1:2, and 2:1, respectively. Simply, 0.01 g Fe_3_O_4_ was dispersed in 60 mL DMF followed by mixing a specific amount of CuNiMn-LDH; the mixture was left at room temperature under mild stirring for 30 min for well dispersion. Then, 0.215 g Co(NO_3_)_2_.6H_2_O and 0.235 g MeIm were added and stirred for 1 h. Thereafter, the mixture solution was dipped into a stainless autoclave and heated for 24 h at 125 °C. Finally, the produced magnetic MOF/LDH composite was washed and dried at 70 °C overnight.

### Characterization

The synthesized catalysts were analyzed via X-ray diffractometer (XRD- MAC Science M03XHF) to examine the crystalline phase. The chemical composition was identified via Fourier transform-infrared spectra (FTIR- Tensor II, Bruker). Moreover, X-ray photoelectron spectroscopy (XPS- Thermo scientific ESCALAB 250Xi VG) was applied to analyze the samples’ elemental composition, whereas Zeta-sizer (ZP-Malvern) was utilized to examine the surface charge. Also, the samples’ morphologies were analyzed by scanning electron microscope and energy dispersive X-ray microscope (SEM, SEM-EDX- JSM-760F). The magnetization of Fe_3_O_4_@ZIF-67/CuNiMn-LDH catalyst was assessed via vibrating sample magnetometer (VSM- Oxford Type 1.2T). The concentrations of the leached metals were determined by an inductively coupled plasma mass spectrometer (ICP-MS, M90, Bruker). The degradation pathways were supposed by Gas chromatography–mass spectrometry (SHIMADZU, GCMS-QP 2010 Ultra).

### Fenton-like experiments

Congo red was utilized as a toxic-waste model for evaluating the catalytic activity of Fe_3_O_4_@ZIF-67/CuNiMn-LDH composite. The degradation experiments are accomplished as follows: under agitation speed 200 rpm, 0.01 g Fe_3_O_4_@ZIF-67/CuNiMn-LDH was added to 20 mL CR solution, and 1 mL H_2_O_2_. The optimum conditions of the catalytic degradation process were defined by studying the controlling parameters like the solution pH which was scrutinized in the range of 2–10. In addition, the influence catalyst dosage by varying the mass of Fe_3_O_4_@ZIF-67/CuNiMn-LDH at the range of 0.005–0.02 g. The influence of the solution temperature was assessed at a temperature ranging from 25 to 55 °C. Also, the impact of the concentration of H_2_O_2_ was tested in the range of 100–500 mg/L, and the initial concentration of CR was in the range of 50–200 mg/L. Furthermore, the catalytic activity of Fe_3_O_4_@ZIF-67/CuNiMn-LDH was assessed toward cationic dyes (MB, MG, and CV) and anionic dyes (MR, MO, and CR). Moreover, the reactive species was defined by quenching test using scavengers such as t-butyl alcohol (TBA) and chloroform. The H_2_O_2_ concentration was detected by applying the potassium titanium (IV) oxalate spectrophotometric method with a wavelength adjusted at 385 nm. During the catalytic degradation process, a 3 mL sample of the solution was withdrawn, and its concentration was determined via UV-vis spectrometer at *λ*_max_ = 500 nm. The CR degradation (%) was calculated from Eq. 2:

2 $$\mathrm{CR}\ \mathrm{degradation}\left(\%\right)=\frac{{\mathrm{C}}_{\mathrm{o}}-{\mathrm{C}}_{\mathrm{t}}}{{\mathrm{C}}_0}\times 100$$

C_o_ symbolizes the initial concentration of CR, and C_t_ expresses the CR concentration at a run time.

### Extraction and analysis of CR degradation products

The degradation of CR dye by Fe_3_O_4_@ZIF-67/CuNiMn-LDH catalyst produces various intermediates which are identified via GC-MS analysis. After completing the degradation reaction of CR, the degraded sample was centrifuged for 10 min. The produced compounds after the CR degradation were extracted using liquid–liquid extraction by equal ratios of ethyl acetate and samples. The extracted organic phase was dried over Na_2_SO_4_, then evaporated till dryness by rotary evaporation. Eventually, the obtained product was dissolved into methanol.

The extracted product was injected into a (0.18 mm diameter × 20 m long, 0.18 mm film thickness) PE-5MS column with initial temperature adjusted at 80 °C for 2 min, then increased by 10 °C min^−1^ until reaching 280 °C followed by holding for 7 min.

### Recycle test

 The reusability of AOP catalysts has an immense spotlight in the actual practical application. The reusability of Fe_3_O_4_@ZIF-67/CuNiMn-LDH catalyst was estimated by five consecutive runs of CR degradation. After each run, Fe_3_O_4_@ZIF-67/CuNiMn-LDH was washed with acetone and dried at 60 °C for 12 h. Then, the regenerated catalyst was examined in the next run.

## Results and discussion

### Characterization of Fe_3_O_4_@ZIF-67/CuNiMn-LDH

#### FTIR

**Fig. 1:**
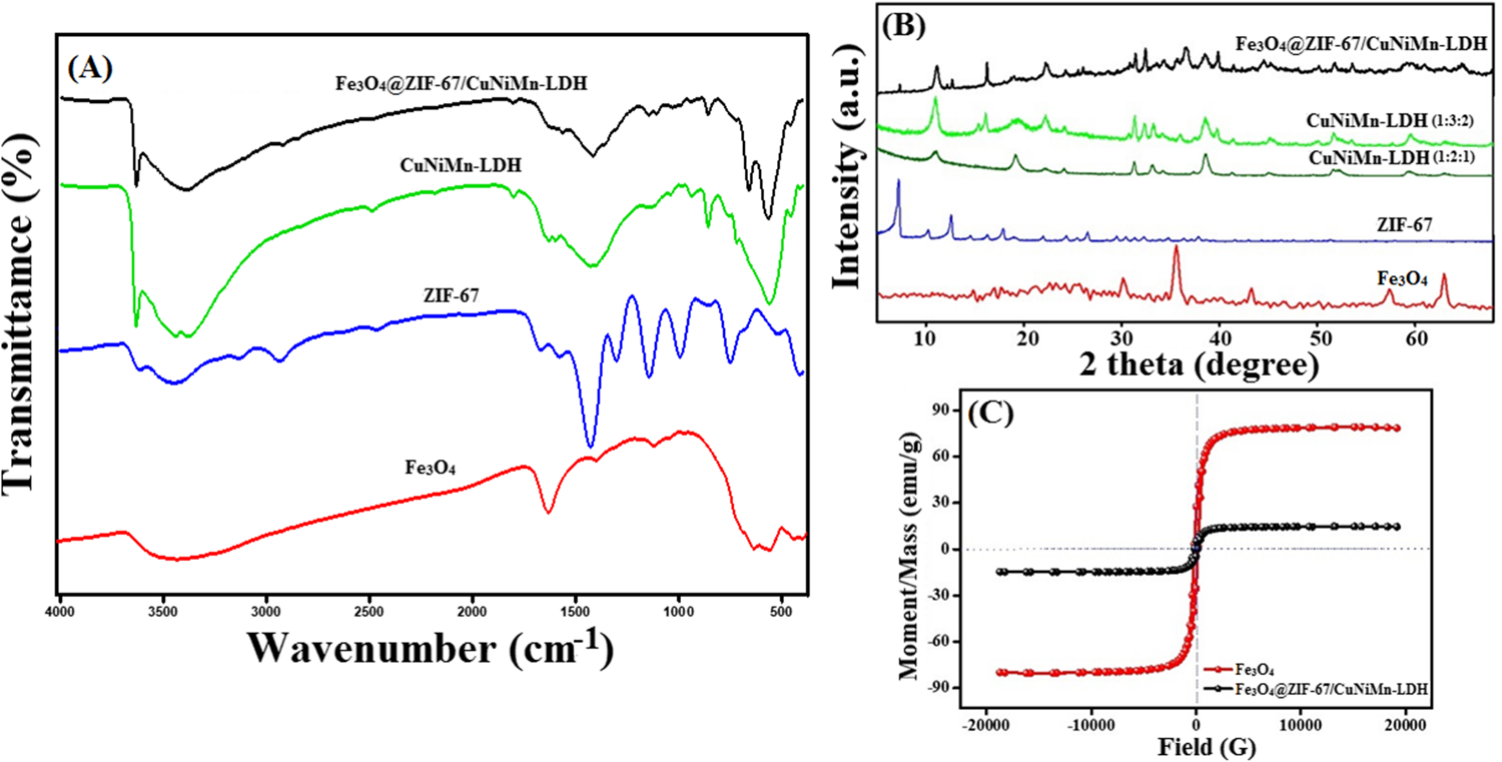
**A** FTIR spectra; **B** XRD patterns of Fe_3_O_4_, ZIF-67, CuNiMn-LDH, and Fe_3_O_4_@ZIF-67/CuNiMn-LDH composite; and **C** VSM of Fe_3_O_4_ and Fe_3_O_4_@ZIF-67/CuNiMn-LDH

Figure 1A reveals the FTIR spectra of Fe_3_O_4_, ZIF-67, CuNiMn-LDH, and Fe_3_O_4_@ZIF-67/CuNiMn-LDH. For Fe_3_O_4_, the peaks at 561 and 1417 cm^−1^ are credited to Fe–O–Fe stretching vibration (Eltaweil et al. 2021). The bands at 892 and 1632 cm^−1^ are assigned to OH vibration and bending modes, respectively. For ZIF-67, the peaks at 2921 and 1565 cm^−1^ are ascribed to stretching aromatic ring and stretching C=N of 2-MeIm, sequentially, while the peaks at 1417, 459, and 994 cm^−1^ are assigned to C=C, Co–N stretching and bending vibration of C–N, respectively (Omer et al. 2021, Wei et al. 2020). For CuNiMn-LDH, the broad band at 3440 cm^−1^ belongs to O–H stretching, and the absorbance peak at 1630 cm^−1^ is attributed to the bending vibration of H_2_O and the interlayer distance of the LDH. Furthermore, the manifestation of the band at 1041 cm^−1^ is ascribed to the Cu–O, while the peaks ranging from 400 to 700 cm^−1^ correspond to the bending and stretching modes of M–O bonds and M–OH, where M is credited to Ni and Mn (Baig et al. 2021, Saghir et al. 2020). FTIR elucidates the formation of Fe_3_O_4_@ZIF-67/CuNiMn-LDH since the characteristic absorbance bands of Fe_3_O_4_, ZIF-67, and CuNiMn-LDH are manifested clearly in the spectrum.

#### XRD

The crystal profiles of Fe_3_O_4_, ZIF-67, CuNiMn-LDH (1:2:1, and 1:3:2), and Fe_3_O_4_@ZIF-67/CuNiMn-LDH were inspected by XRD as depicted in Fig. 1B. The XRD profile shows the characteristic diffraction peaks of Fe_3_O_4_ at 2θ = 30.1°, 35.8°, 43.1°, 57.01°, and 62.8° which are accompanied by (220), (311), (400), (511), and (440) planes, sequentially (Chen et al. 2021). These characteristic peaks are well consistent with the standard PDF card (JCPDS No. 03-0863). Furthermore, the XRD profile of ZIF-67 depicts sharp peaks at 7.4°, 10.4°, 12.8°, 14.5°, 18.0°, 24.5°, and 26.8° which corresponded to (011), (002), (112), (022), (222), (233), and (134) planes, respectively (Omer et al. 2021). The XRD profile of CuNiMn-LDH (1:2:1) shows a prominent decline in the crystallinity when the Cu ratio elevated in the LDH, owing to the influence of Jahn–Teller distortion, as Cu^2+^ installed a distorted Cu complex octahedral (Zhu et al. 2022). On the other hand, the XRD pattern of CuNiMn-LDH (1:3:2) elucidated its high crystallinity, revealing the diffraction peaks with high intensity at 11.06°, 22.2°, 32.6°, 38.5°, 47.8°, 59.5°, and 62.9°, corresponding by (003), (006), (101), (015), (018), (110), and (113) planes, respectively. Thence, the ratio of 1:3:2 was chosen as the optimal metal cations ratio to fabricate CuNiMn-LDH. The XRD profile of Fe_3_O_4_@ZIF-67/CuNiMn-LDH composite reveals the discriminative diffraction peaks of Fe_3_O_4_, ZIF-67, and CuNiMn-LDH peaks, but with lower peaks intensity compared to the pristine phase, emphasizing the successful combination between them.

#### VSM

VSM hysteresis loops (Fig. 1C) exhibit the soft ferromagnetic nature of Fe_3_O_4_ and Fe_3_O_4_@ZIF-67/CuNiMn-LDH in which the coercivity value of both magnetic samples exceeded 20 G. Furthermore, it was recorded a noticeable diminution in the saturation magnetization of Fe_3_O_4_@ZIF-67/CuNiMn-LDH (12.5 emu/g) compared to the pristine the Fe_3_O_4_ (78.5 emu/g). Such a finding is most likely due to the non-magnetic nature of both ZIF-67 and CuNiMn-LDH as well as the low proportion of Fe_3_O_4_ in the as-fabricated composite.

#### XPS

**Fig. 2:**
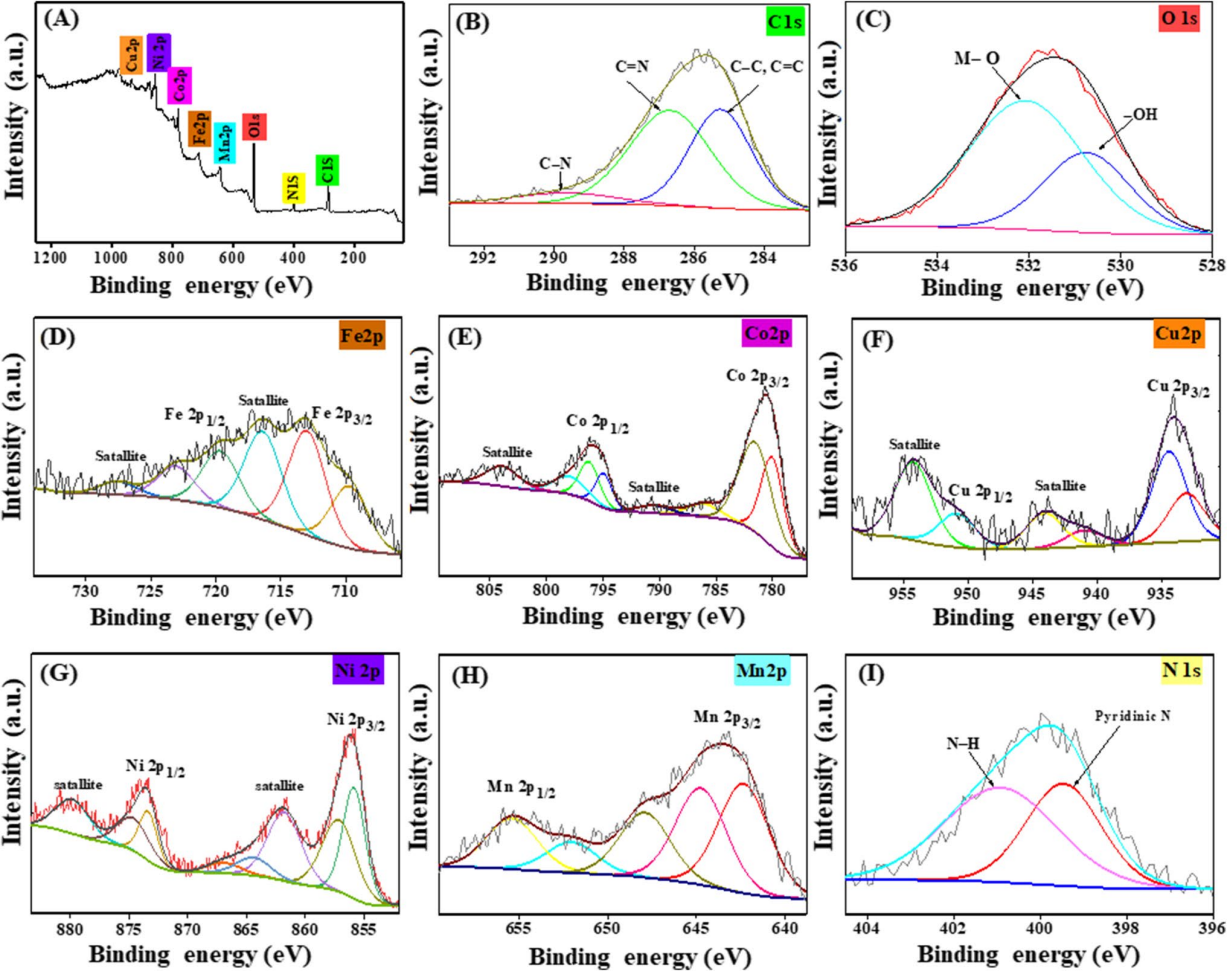
XPS spectra of Fe_3_O_4_@ZIF-67/CuNiMn-LDH **A** survey, **B** C 1s, **C** O1s, **D** Fe 2p, **E** Co 2p, **F** Cu 2p, **G** Ni 2p, **H** Mn 2P, and **I** N 1s

To verify the composition of Fe_3_O_4_@ZIF-67/CuNiMn-LDH, XPS analysis was applied. The survey spectrum reveals the main elements of the composite Fe, C, N, Co, O, Cu, Ni, and Mn as exhibited in Fig. 2A. The C1s spectrum (Fig. 2B) points out the peaks at 285.24 and 286.69 eV which can be credited to C–C/C=C and C=N, respectively, and the characteristic peak of the C–N bond appeared at 289.67 eV (Zhang et al. 2021a). The O1s spectrum (Fig. 2C) elucidates a peak at 531.78 eV which corresponds to the surface hydroxyl group, and the peak at 530.48 eV belongs to the M-O bonds (Wu et al. 2022a, Zhao et al. 2018). Furthermore, the peaks of Fe^2+^ 2p_3/2_, Fe^3+^ 2p_3/2_, Fe^2+^ 2p_1/2_, and Fe^3+^ 2p_1/2_ are detected nearby 709.70, 712.99, 719.65, and 723.06 eV, respectively (Fig. 2D). The distinguishing peaks of Co 2p_3/2_; Co^2+^ (octahedral site), Co^2+^ (tetrahedral site), and Co^3+^ (octahedral site) were spotted at 780.03, 781.54, and 786.10 eV, respectively, while the characteristic peaks of Co 2p_1/2_; Co^2+^ (octahedral site), Co^2+^ (tetrahedral site), and Co^3+^ (octahedral site) appeared at 794.94, 795.96, and 797.10 eV, respectively. Also, there are two peaks at 803.92 and 790.48 eV that are assigned to satellite peaks (Fig. 2E) (Khan et al. 2020, Yu et al. 2018). For the Cu 2p spectrum (Fig. 2F), the peaks at 934.39 and 933.06 eV are assigned to Cu^2+^ 2p_1/2_ and Cu^+^ 2p_1/2_, respectively, while the peaks at 944.09 and 950.85 eV are ascribed to Cu^+^ 2p_3/2_ and Cu^2+^ 2p_3/2_, respectively. In addition, the existence of the two satellite peaks is at 940.99 and 954.20 eV (Wang et al. 2018, Wu et al. 2022a). Additionally, the spectrum of Ni 2p illustrates the peaks at 855.86, 873.27, 873.27, and 873.88 eV, which are referred to Ni^2+^ 2p_3/2_, Ni^3+^ 2p_1/2_, Ni^2+^ 2p_1/2_, and Ni^3+^ 2p_1/2_, respectively (Fig. 2G) (Yu et al. 2018). For Mn 2p spectrum (Fig. 2H), the Mn 2p_3/2_ peaks spotted at 642.34 eV, 644.74 eV, and 647.89 eV belong to Mn^2+^, Mn^3+^, and Mn^4+^, respectively (Wu et al. 2022b), while the Mn 2p_1/2_ peaks located at 652.04 eV and 655.39 eV are characterized to Mn^2+^ and Mn^3+^, respectively (Chen et al. 2021). Moreover, the discriminative peak of N-H and N of the imidazole ring at 400.23 and 399.19 eV is illustrated in Fig. 2I (Wei et al. 2020).

#### SEM 

**Fig. 3:**
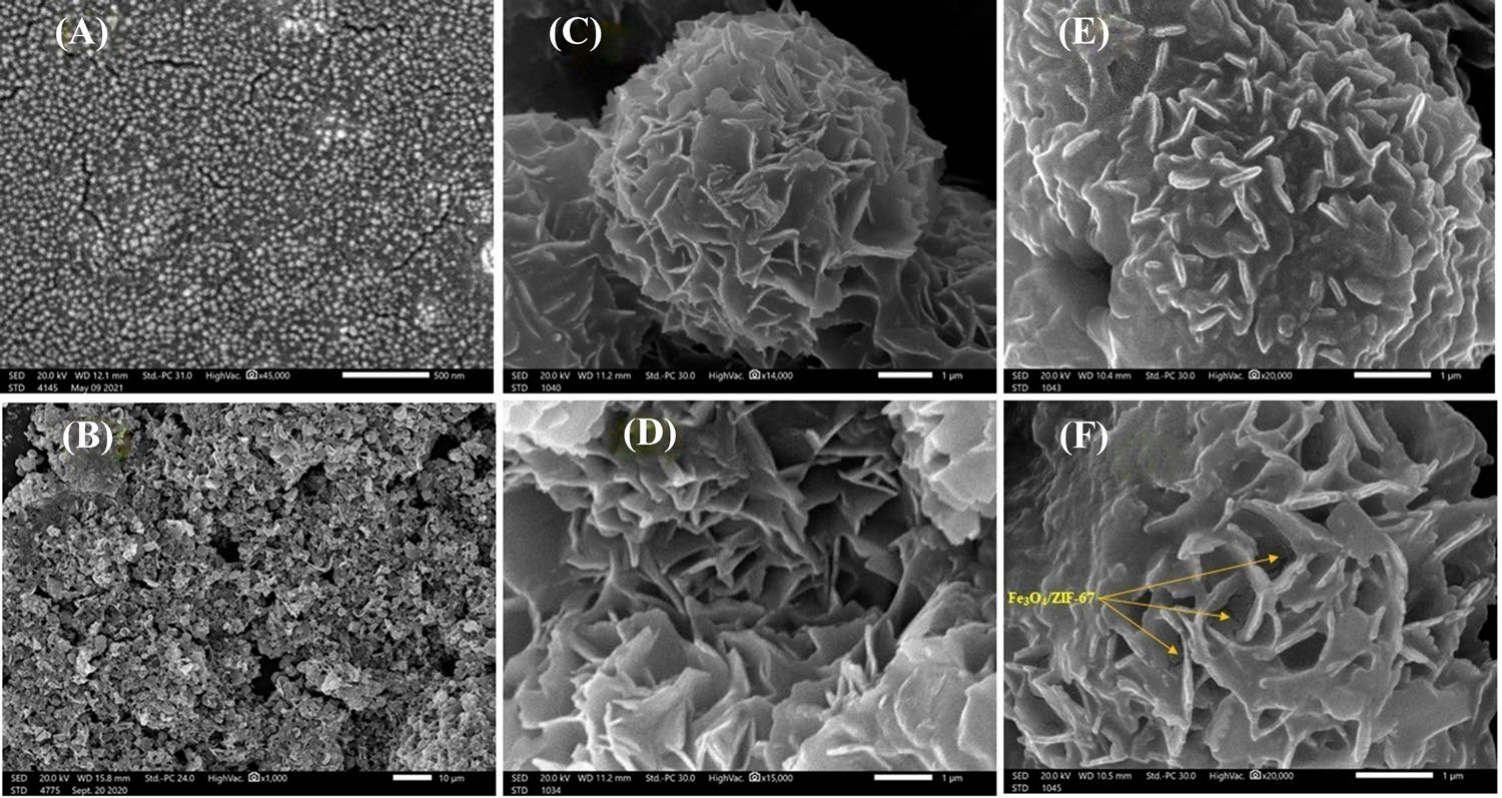
The SEM images; **A** Fe_3_O_4_, **B** ZIF-67, **C** and **D** CuNiMn-LDH, and **E** and **F** Fe_3_O_4_@ZIF-67/CuNiMn-LDH

For inspection of the catalysts’ morphology, SEM was used as elucidated in Fig. 3. The SEM of Fe_3_O_4_ (Fig. 3A) points out a smooth spherical shape in nano size, forming non-uniform agglomerates. The SEM image (Fig. 3B) reveals an irregular morphology of ZIF-67, while Fig. 3C and D figure out the flower-like of CuNiMn-LDH with porous spheres morphology comprised of stacked ultrathin sheets. Moreover, the SEM image of Fe_3_O_4_@ZIF-67/CuNiMn-LDH (Fig. 3E, F) shows some distortion in the flower structure of CuNiMn-LDH, which is most likely due to the random allocation of ZIF-67 on the surface of CuNiMn-LDH, in addition to adherence of Fe_3_O_4_ nanoparticles. Also, the SEM image clarifies the existence of Fe_3_O_4_/ZIF-67 between the ultrathin sheets of CuNiMn-LDH.

### Catalytic activity of Fe_3_O_4_@ZIF-67/CuNiMn-LDH

#### Comparison test

**Fig. 4:**
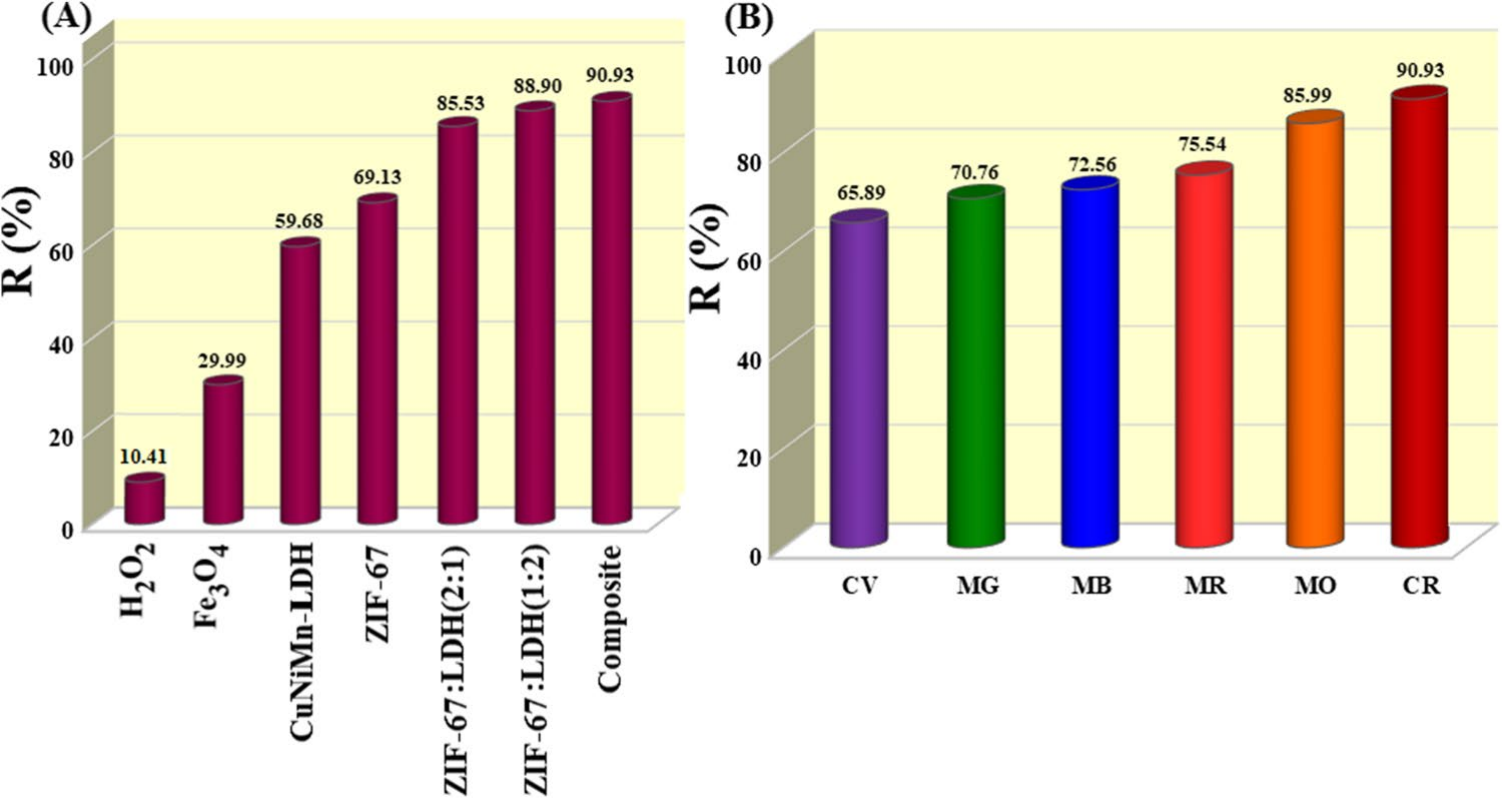
**A** Comparison test between H_2_O_2_, Fe_3_O_4_, CuNiMn-LDH, ZIF-67, and Fe_3_O_4_@ZIF-67/CuNiMn-LDH composites (*t* = 30 min, H_2_O_2_ concentration = 500 mg/L, *T* = 25 °C, catalyst dosage = 0.01 g, and the CR initial concentration = 50 mg/L), and **B** the degradation activity of Fe_3_O_4_@ZIF-67/CuNiMn-LDH toward different dyes (*t*= 30 min, H_2_O_2_ concentration = 500 mg/L, *T* = 25 °C, catalyst dosage = 0.01 g, and the dyes initial concentration = 50 mg/L)

To scrutinize the synergistic effect between the pure components of Fe_3_O_4_@ZIF-67/CuNiMn-LDH composite, a comparison test was executed between Fe_3_O_4_, ZIF-67, CuNiMn-LDH, and Fe_3_O_4_@ZIF-67/Cu Ni Mn-LDH composites. As exhibited in Fig. 4A, the degradation percent of CR by H_2_O_2_, Fe_3_O_4_, ZIF-67, CuNiMn-LDH, and Fe_3_O_4_@ZIF-67/Cu Ni Mn-LDH reached 10.41, 29.99, 69.13, 59.68, and 90.90%, respectively. The superior degradation percent of Fe_3_O_4_@ZIF-67/CuNiMn-LDH owes to the synergistic effect between Fe, Cu, Co, Ni, and Mn which boosts the redox cycle of Fe^3+^/Fe^2+^, Cu^2+^/Cu^+^, Co^3+^/Co^2+^, Ni^2+^/Ni^3+^, and Mn^3+^/Mn^4+^ (Wang et al. 2018, Wu et al. 2022a). Furthermore, different ratios of MOF: LDH in the catalyst were investigated; where, the degradation efficiency attained 85.5, 90.9, and 88.9% for MOF: LDH with ratios 1:2, 1:1, and 2:1, respectively. Consequently, the composite which contains the equal ratio between MOF and LDH was chosen for the rest experiments.

#### Fenton-like degradation of various dyes

For further investigation of the catalytic degradation activity of Fe_3_O_4_@ZIF-67/CuNiMn-LDH, it was tested in Fenton-like degradation of various organic dyes, CV, MG, MB, MR, and MO. Furthermore, the degradation efficiency of these dyes was compared to that of CR to evince the selectivity of Fe_3_O_4_@ZIF-67/CuNiMn-LDH toward CR (Fig. 4B). The catalytic activity test exhibited that the degradation efficiencies of CV, MG, MB, MR, MO, and CR by the Fe_3_O_4_@ZIF-67/CuNiMn-LDH/H_2_O_2_ system were 65.86, 70.76, 72.56, 75.54, 85.99, and 90.90%, respectively. Such results imply the propitious activity of the Fe_3_O_4_@ZIF-67/CuNiMn-LDH/H_2_O_2_ system toward the cationic and anionic dyes, but more selective toward the anionic dyes. Importantly, the chemical structure of these dyes plays a vital role in facilitating the degradation process; CR, MR, and MO are functionalized as azo dyes owing to having chromophore centers (-N=N-), which it be easily attacked by ^•^OH radicals due to the existence of the easy-cleavage π-bond. On the other hand, the degradation reactions of MB, MG, and CV take place on the nitrogen-atoms bond of their inner ring. Since the presence of the electron-donating (-N(CH_3_)_2_) group notably increased the electron density of nitrogen-atoms that bond to the benzene ring, thereby, the inner rings of MB, MG, and CV are smoothly broken (Zhao et al. 2018). In light of these results, CR was picked out as a model of anionic dye to evaluate the catalytic activity of Fe_3_O_4_@ZIF-67/CuNiMn-LDH.

### Effect of the controlling parameters on the Fenton-like degradation of CR

#### The influence of pH

**Fig. 5:**
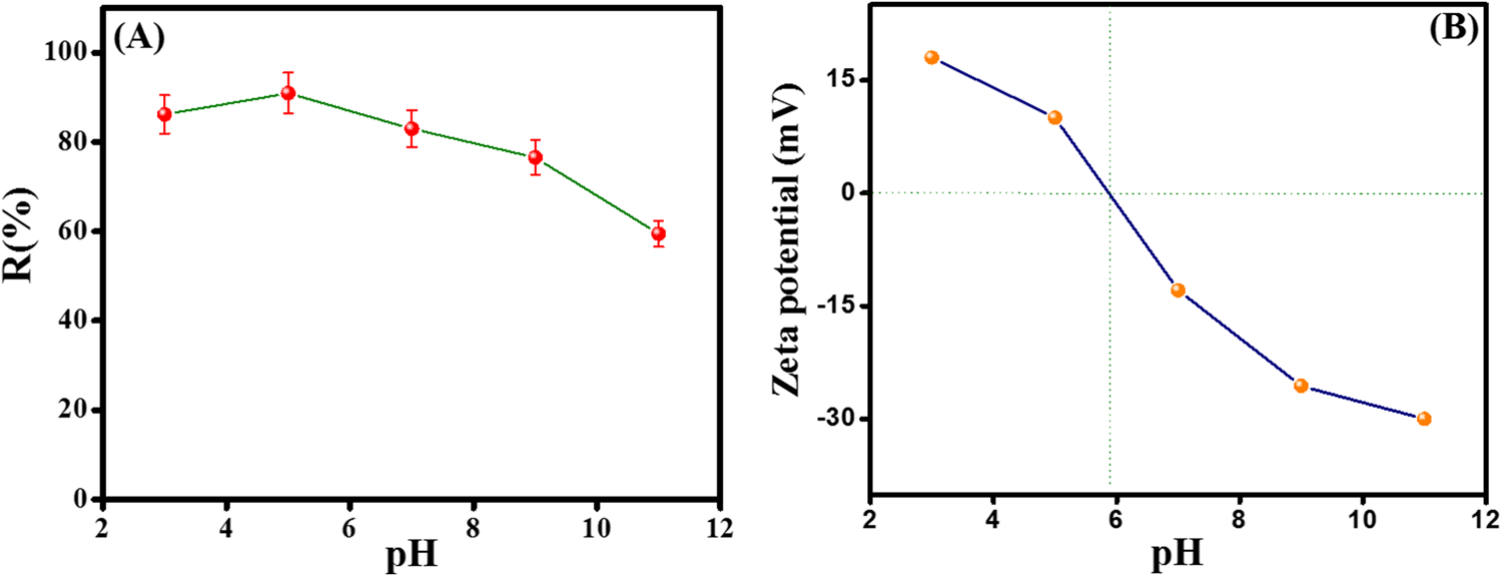
**A** The impact of pH on the Fenton-like degradation of CR (catalyst dosage= 0.01 g, H_2_O_2_ concentration = 500 mg/L, *T*= 25 °C, and CR initial concentration= 50 mg/L), and **B** zeta potential versus pH for Fe_3_O_4_@ZIF-67/CuNiMn-LDH

Figure 5A illustrated that the catalytic activity of Fe_3_O_4_@ZIF-67/CuNiMn-LDH is mainly affected by altering the initial pH value of the CR solution at a pH ranging from 3 to 11. It was deduced that the maximum degradation efficiency of CR by Fe_3_O_4_@ZIF-67/CuNiMn-LDH was 90.9% at *pH*= 5 within 30 min. The results depict that the CR degradation under weak acidic or near neutral conditions was much better than in strongly acidic conditions (*pH*= 3) or strong basic conditions (*pH*= 11). At strongly acidic conditions, the produced ^•^OH radicals may be scavenged via H^+^ causing a striking decrease in the degradation efficiency of CR (Eqs. 3, 4). While the remarkable diminution in the degradation efficiency of CR under strongly basic conditions can be ascribed to the formation of the highly nucleophilic hydroperoxy anions (OOH^−^) that possesses higher affinity towards metal ions (M= Co, Fe, Cu, Ni, and Mn) than H_2_O_2_ (Eqs. 5, 6) (Shen et al. 2017), in addition to the possibility of auto-decomposition of H_2_O_2_ in the strongly basic medium as clarified in Eq. 7 (Shi et al. 2018). On the other hand, ZP measurements (Fig. 5B) investigated that the pH_zpc_ of Fe_3_O_4_@ZIF-67/CuNiMn-LDH was ~5.8. Thence, the surface of Fe_3_O_4_@ZIF-67/CuNiMn-LDH charges with positive charges when *pH* < 5.8, which enhances the degradation efficiency owing to the electrostatic attraction between the anionic CR and Fe_3_O_4_@ZIF-67/CuNiMn-LDH. While at *pH* > 5.8, the electrostatic repulsion forces between CR and the negatively charged surface of Fe_3_O_4_@ZIF-67/CuNiMn-LDH dwindle the CR degradation efficiency.

3 $${}{}^{\bullet}\mathrm{O}\mathrm{H}+\mathrm{RH}\to {\mathrm{R}}^{\bullet }+{\mathrm{H}}_2\mathrm{O}$$

4 $${\mathrm{R}}^{\bullet }+\mathrm{OH}\to \mathrm{ROH}$$

5 $${\mathrm{H}}_2{\mathrm{O}}_2+\mathrm{O}{\mathrm{H}}^{-}\to \mathrm{OO}{\mathrm{H}}^{-}+{\mathrm{H}}_2\mathrm{O}$$

6 $$\mathrm{M}+\mathrm{OO}{\mathrm{H}}^{-}\to \mathrm{M}\ldots {}{}^{\bullet}\mathrm{O}\mathrm{O}\mathrm{H}$$

7 $$2{\mathrm{H}}_2{\mathrm{O}}_2\to {\mathrm{O}}_2+2{\mathrm{H}}_2\mathrm{O}$$

#### The influence of catalyst dosage

**Fig. 6:**
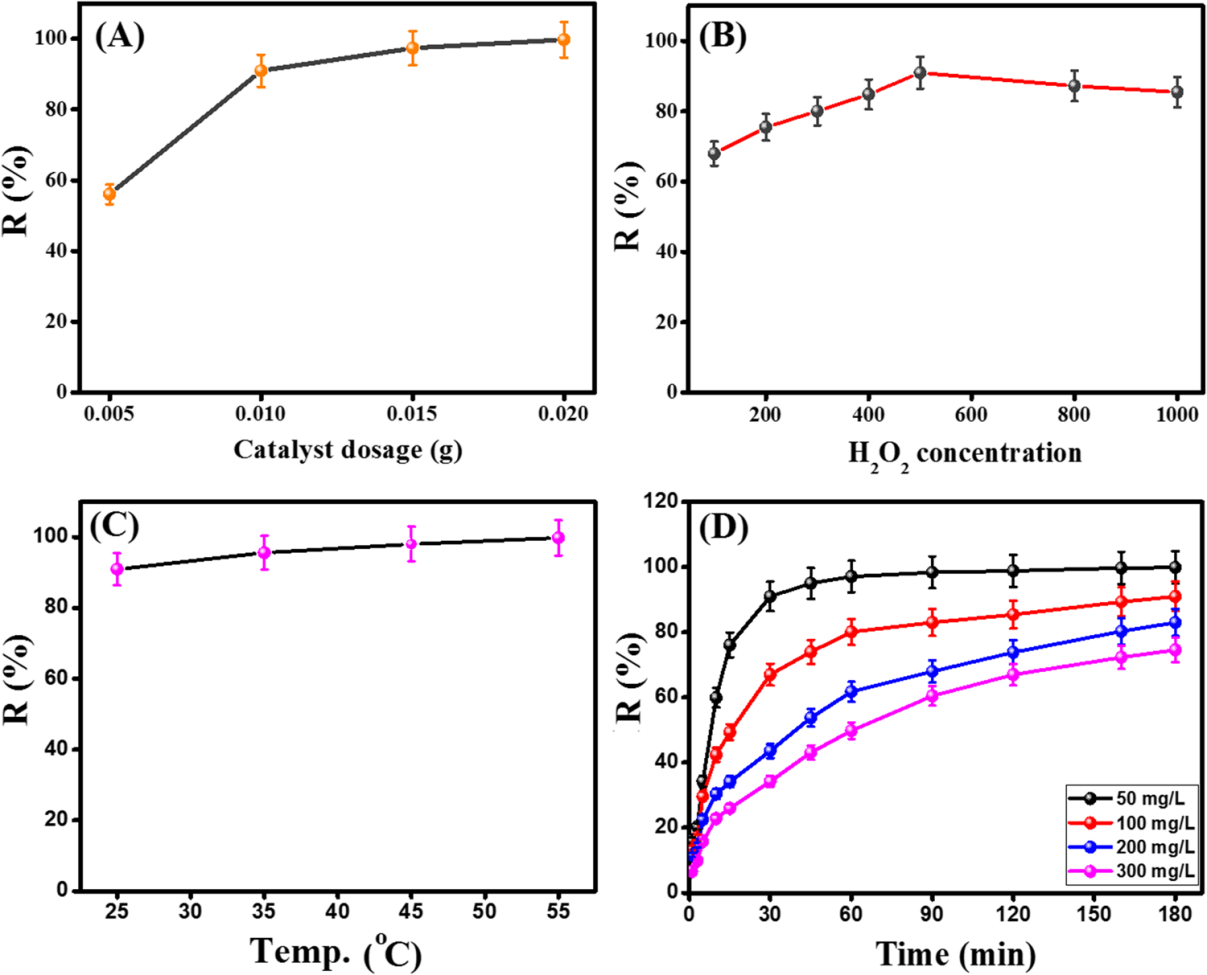
The controlling factors on the Fenton-like degradation of CR; **A** the catalyst dosage (*pH*= 5, *T*= 25 °C, H_2_O_2_ concentration = 500 mg/L, and CR concentration= 50 mg/L), **B** the H_2_O_2_ concentration (*pH*=5, catalyst dosage= 0.01 g, CR concentration= 50 mg/L, and *T*= 25 °C), **C** the temperature (*pH*=5, H_2_O_2_ concentration = 500 mg/L, catalyst dosage= 0.01 g, and CR initial concentration= 50 mg/L), and **D** the CR initial concentration (*T*= 25 °C, *pH*=5, H_2_O_2_ concentration = 500 mg/L, and catalyst dosage= 0.01 g)

Figure 6A represents the impact of varying the Fe_3_O_4_@ZIF-67/CuNiMn-LDH dosages on the CR degradation efficacy. It is obvious that the raising in Fe_3_O_4_@ZIF-67/CuNiMn-LDH dosage from 0.005 to 0.02 g boosts the CR degradation efficiency from 56.5 to 99.6%, which is most likely due to the generation of more free radicals in the presence of excess amounts of the catalyst. However, 0.01 g was chosen as the optimal dosage of Fe_3_O_4_@ZIF-67/CuNiMn-LDH for economic and environmental reasons.

#### The influence of H_2_O_2_ concentration

The H_2_O_2_ concentration is one of the controlling parameters of Fenton-like reaction since H_2_O_2_ is the main source of ^•^OH radicals. Figure 6B exhibits the substantial impact of the H_2_O_2_ concentration on the CR degradation aptitude. It was observed that the CR degradation efficiency notably improved from 67.9 to 90.9% by raising the H_2_O_2_ concentration from 100 to 500 mg/L, respectively, owing to the increase in the generated ^•^OH radicals. Nevertheless, the further increase in the H_2_O_2_ concentration over 500 mg/L declines the CR degradation efficiency since the much more H_2_O_2_ concentrations may act as scavengers (Eqs. 8, 9), agreeing with Xin et al. study (Xin et al. 2021).

8 $${\mathrm{H}}_2{\mathrm{O}}_2+{}{}^{\bullet}\mathrm{O}\mathrm{H}\to \mathrm{HO}{\mathrm{O}}^{\bullet }+{\mathrm{H}}_2\mathrm{O}$$

9 $$\mathrm{HO}{\mathrm{O}}^{\bullet }+{}{}^{\bullet}\mathrm{O}\mathrm{H}\to {\mathrm{H}}_2\mathrm{O}+{\mathrm{O}}_2$$

#### The influence of temperature

The impact of the reaction temperature on the CR degradation by Fe_3_O_4_@ZIF-67/CuNiMn-LDH/H_2_O_2_ system was scrutinized as represented in Fig. 6C. The experimental results point out that the increase in the temperature from 25 to 55 °C increases the CR degradation percentage reaching ~100%. This thermal behavior is most probably due to the prompting of the interaction between Fe_3_O_4_@ZIF-67/CuNiMn-LDH and H_2_O_2_ at higher temperatures, producing more reactive ^•^OH species, which directly enhances the efficiency of the CR degradation process (Luo et al. 2020).

#### The influence of the initial CR concentration

Figure 6D displays a reduction in the CR degradation efficiency by the Fe_3_O_4_@ZIF-67/CuNiMn-LDH/ H_2_O_2_ system with increasing the initial concentration of CR. For the initial CR concentrations of 50, 100, 200, and 300 mg/L, the degradation efficiency within 120 min attained 98.8, 89.4, 73.8, and 66.9%, respectively. This decline in the CR degradation percent can be explained by the insufficient accessible active sites on the Fe_3_O_4_@ZIF-67/CuNiMn-LDH surface for such high concentrations of CR (Wu et al. 2022a). Besides, the blocking of the active sites by the high concentration of the adsorbed CR leads to fewer production of ^•^OH radicals (Hassani et al. 2018).

### Kinetic study

 For investigating the CR degradation kinetics, the experimental data were inspected by pseudo-first-order kinetic model (Eq. 10).

10 $$-\ln \left(\frac{C}{C_o}\right)= kt$$

C_o_ and C are the initial concentration and the concentration at a certain time of the CR dye, respectively, k (min^−1^) is the rate constant, and t is reaction time (min).

**Fig. 7:**
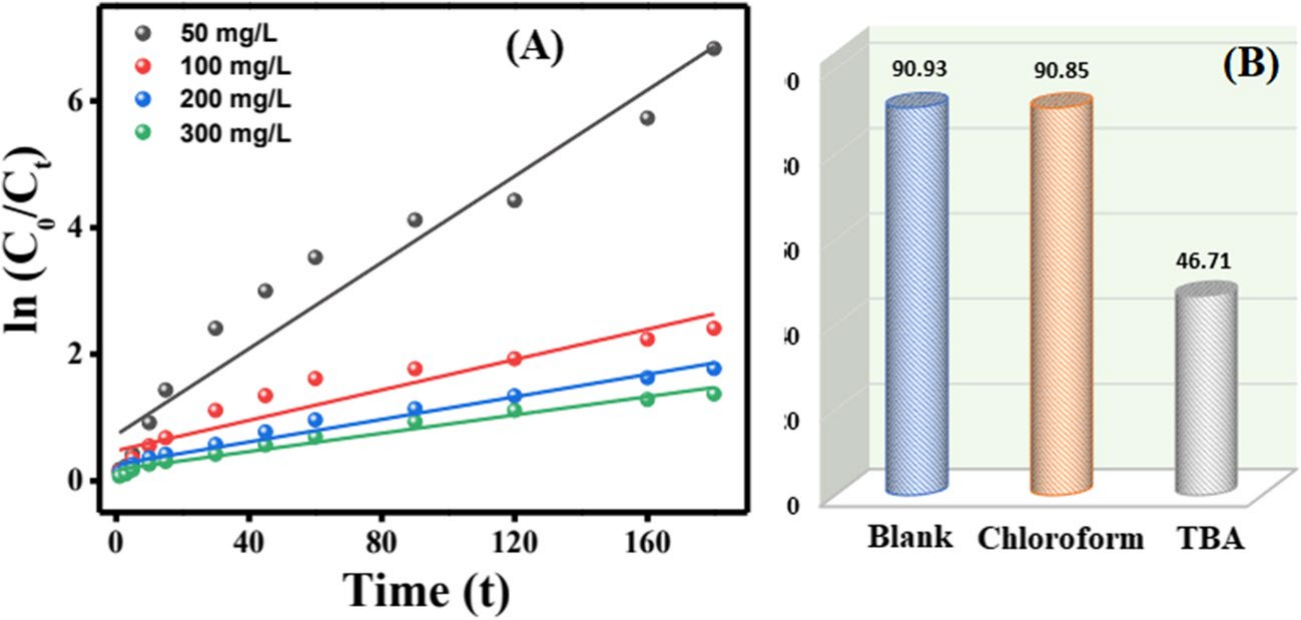
**A** Pseudo-first-order kinetic plot and **B** quenching test of the CR degradation via the H_2_O_2_ activation by Fe_3_O_4_@ZIF-67/CuNiMn-LDH

Figure 7A represents a plot of -ln (C/C_o_) against time that was applied at different CR concentrations of 50, 100, 200, and 300 mg/L. Such a linear graph and the calculated R^2^ inferred that the degradation of CR at these concentrations fitted pseudo-first-order kinetic model. The R^2^ and k values are summarized in Table S1.

### Identification of reactive species

For detecting the controlling ROS on the Fenton-like degradation of CR, a quenching test was implemented with TBA and chloroform as scavengers for ^•^OH and ^•^O_2_^−^ species, respectively [59]. It was deduced that the CR degradation efficiency significantly dwindled from 90.9 to 46.711% in the existence of TBA, while there was no significant decrease in degradation rate when chloroform was added (Fig. 7B). This finding infers the predominance of ^•^OH radical in the CR degradation process over Fe_3_O_4_@ZIF-67/CuNiMn-LDH catalyst. On the contrary, ^•^O_2_^−^ species have an ineffective role in the degradation process.

### Degradation mechanism

In general, the activation of H_2_O_2_ occurs via two pathways; the first one is the surface oxygen vacancy pathway that takes place via the surface reaction of the oxygen vacancies (OV) on the oxide surface, producing O_2_ species (Eqs. 11, 12), while the radical reaction is the second pathway for the H_2_O_2_ activation via the reaction with metal ion species that generate reactive oxygen radicals (Costa et al. 2006).

11 $$\mathrm{O}\mathrm{V}+{\mathrm{H}}_2{\mathrm{O}}_2\to \mathrm{O}\mathrm{V}.\ldots \mathrm{O}+{\mathrm{H}}_2\mathrm{O}$$

12 $$\mathrm{O}\mathrm{V}.\ldots \mathrm{O}+{\mathrm{H}}_2{\mathrm{O}}_2\to \mathrm{O}\mathrm{V}+{\mathrm{H}}_2\mathrm{O}+{\mathrm{O}}_2$$

Based on the results of the quenching test, it was deduced that the Fenton-like degradation of CR over Fe_3_O_4_@ZIF-67/CuNiMn-LDH catalyst proceeds via the radical reaction pathway since the presence of radical scavengers reduced the degradation efficiency.

**Fig. 8:**
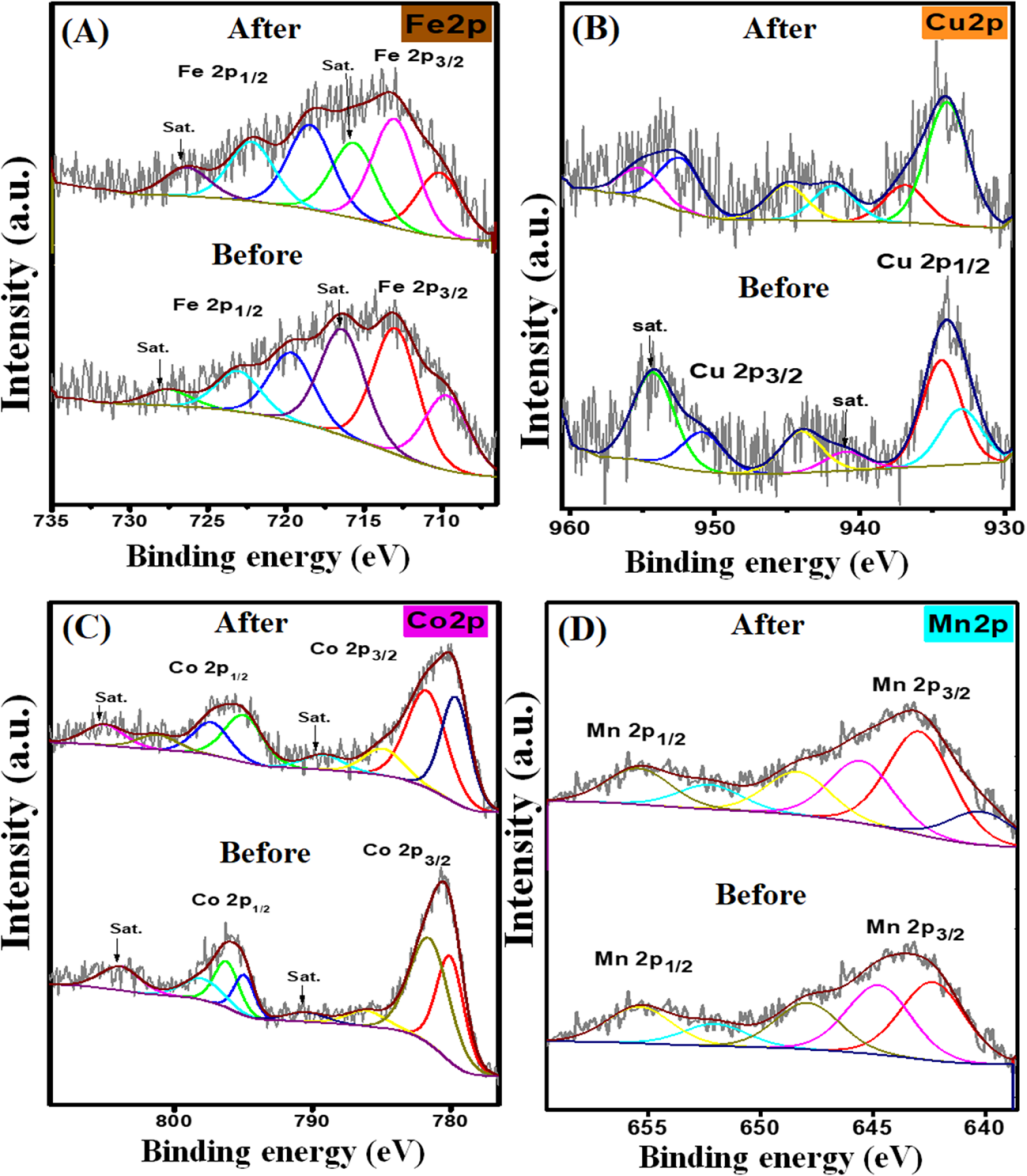
XPS of Fe_3_O_4_@ZIF-67/CuNiMn-LDH before and after the CR degradation; **A** Fe2p, **B** Cu2p, **C** Co2p, and **D** Mn2p

To propose the CR degradation mechanism via the H_2_O_2_ activation by Fe_3_O_4_@ZIF-67/CuNiMn-LDH, the XPS spectra of the catalyst before and after the degradation reaction were thoroughly inspected. The Fe 2p spectra of Fe_3_O_4_@ZIF-67/CuNiMn-LDH catalyst (Fig. 8A) reveal a diminution in the ratio of Fe^3+^/Fe^2+^ from 1.164 to 0.641 after the degradation reaction, accompanied by a slight shift in Fe 2p_3/2_ from 709.70 eV and 712.99 eV to 710.15 eV and 713.04 eV, respectively, inferring the involvement of Fe species in the catalytic reaction where Fe^2+^ activates H_2_O_2_ following Haber–Weiss mechanism (Eqs. 13, 14) (Zhang et al. 2021b). Furthermore, the ability of Cu^+^ to cleave –N=N– of azo dyes by Sandmeyer reaction results in the direct degradation of azo dyes to carbon radicals (Eq. 15) (Shen et al. 2017). Figure 8B exhibits a slight shifting in the related peaks to Cu 2p after the CR degradation with a decline in the Cu^2+^/Cu^+^ ratio from 1.43 to 0.928, suggesting the participation of Cu^+^ in the H_2_O_2_ activation (Eq. 16). It was proposed that Cu^+^ could be regenerated via the reaction of Cu^2+^ with Ni^2+^ (Eq. 17) since the metal-oxo-metal bridge allows the electron transfer from Ni^2+^ to Cu^2+^ (Wang et al. 2018). Notably, the redox potential of Fe^3+^/Fe^2+^ (0.77 V) is higher than that of Cu^2+^/Cu^+^ (0.15 V), thereby the reduction of Fe^3+^ by Cu^+^ to regenerate Fe^2+^ was thermodynamically more preferable as elucidated in Eq. 18 (Luo et al. 2020). The Co 2p spectra (Fig. 8C) point out a diminution in the ratio of Co^3+^/Co^2+^ from 0.289 to 0.224 with a slight shift in Co 2p_3/2_ peaks since Co^2+^ and Co^3+^ shifted from 780.03 eV and 786.10 eV to 781.74 eV and 784.86 eV, respectively, suggesting the contribution of Co^2+^ to the activation of H_2_O_2_ as clarified in Eq. 19. In addition to the shifting in the belonging peaks to Co 2p_1/2_ in which Co^2+^ octahedral and Co^2+^ tetrahedral shifted from 794.94 eV and 795.96 eV to 797.31 eV and 794.98 eV, respectively. Interestingly, the reduction of Co^3+^ via Fe^2+^ and Cu^+^ was predicted since Co^3+/^Co^2+^ redox potential (1.808 V) is significantly higher than Fe^3+^/Fe^2+^ and Cu^2+^/Cu^+^; therefore, Co^2+^ is easily recovered by Fe^2+^ and Cu^+^ (Eqs. 20, 21). The Mn 2p spectra (Fig. 8D) figure out that the ratio of Mn^3+^/Mn^2+^ elevated from 1.05 to 1.37, verifying that a high amount of Mn^2+^ converted to Mn^3+^ during the CR degradation (Eq. 22). Furthermore, it was noticed that Mn^4+^ 2p_3/2_ slightly shifted from 647.89 eV to 655.42 eV with a decline in the atomic percent from 18.40 to 12.01% implies the reduction of Mn^4+^ to Mn^3+^, agreeing with Zhao et al. study (Zhao et al. 2018).

Notably, the formed redox cycle in Fe_3_O_4_@ZIF-67/CuNiMn-LDH is continuous since the metal species could regenerate each other based on their redox potentials. In addition, H_2_O_2_ could be further decomposed by ^•^OH radicals as elucidated in Eq. 23 and generates the unstable hydroperoxyl radical that assists in the recovery M^2+^ (Eq. 24) (Šuligoj et al. 2020).

On the other hand, the CR adsorption mechanism onto Fe_3_O_4_@ZIF-67/CuNiMn-LDH proceeded via the coordination bonds between the transition metal-containing catalyst and the sulfate and amine groups of CR. This suggestion was confirmed throughout the shifting of the XPS peaks of the metals. In addition to the electrostatic interactions between the positively charged Fe_3_O_4_@ZIF-67/CuNiMn-LDH (pHzpc ~5.8) and the anionic CR molecules could contribute to the adsorption mechanism of CR. Furthermore, the oxygenated groups on the Fe_3_O_4_@ZIF-67/CuNiMn-LDH surface could interact with the hydrogen atoms on CR molecules via H-bonding. In addition, the H-bonds could be formed between hydrogen atoms of Fe_3_O_4_@ZIF-67/CuNiMn-LDH and sulfate groups of CR.

**Fig. 9:**
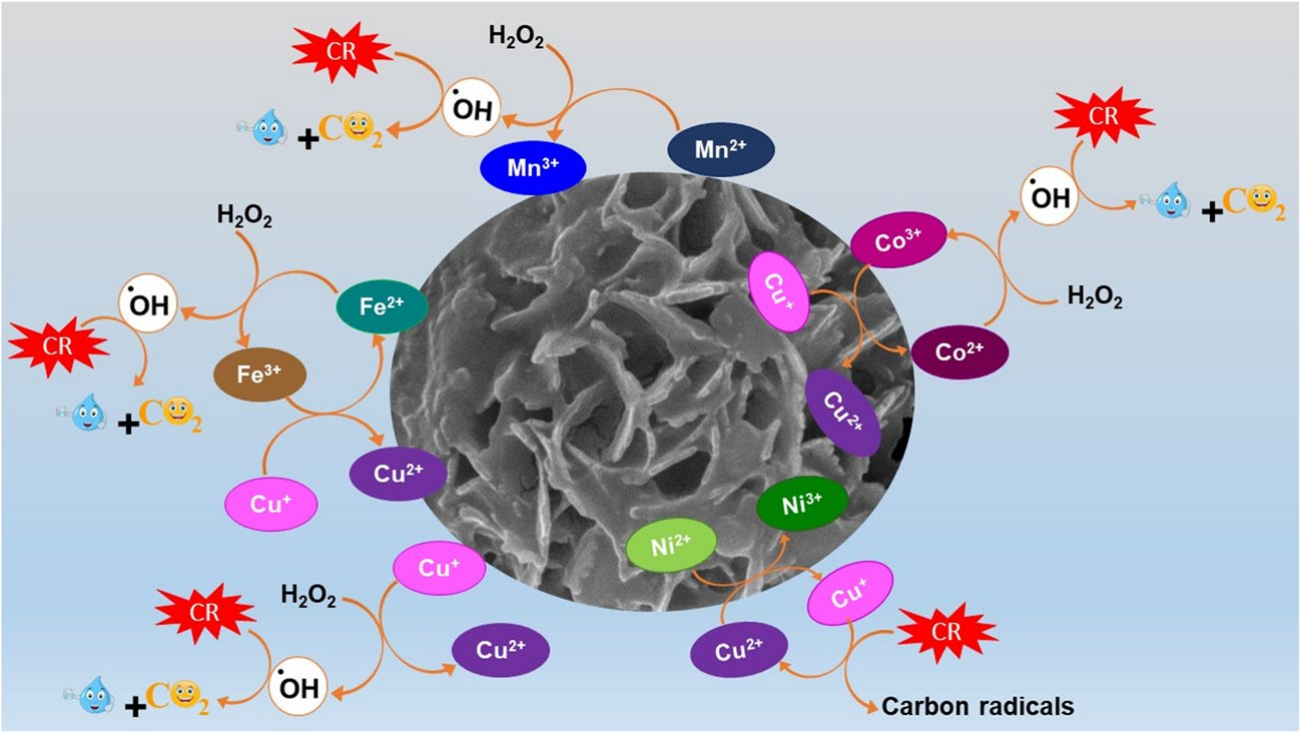
Simple diagram represents the Fenton-like degradation of CR by Fe_3_O_4_@ZIF-67/CuNiMn-LDH/H_2_O_2_ system

In one word, Fe_3_O_4_@ZIF-67/CuNiMn-LDH catalyst possesses a wide redox cycle consisting of Fe^2+^/Fe^3+^, Co^2+^/Co^3+^, Ni^2+^/Ni^3+^, Mn^2+^/Mn^3+^, and Cu^+^/Cu^2+^ (Fig. 9) that provides electrons-rich degradation medium, facilitating the H_2_O_2_ activation and producing abundant ^•^OH, thus could degrade CR with a propitious efficiency (Eq. 25).

13 $${\mathrm{Fe}}^{2+}+{\mathrm{H}}_2{\mathrm{O}}_2\to {\mathrm{Fe}}^{3+}+{}{}^{\bullet}\mathrm{O}\mathrm{H}+\mathrm{O}{\mathrm{H}}^{-}$$

14 $${\mathrm{Fe}}^{3+}+{\mathrm{H}}_2{\mathrm{O}}_2\to {\mathrm{Fe}}^{2+}+{{}{}^{\bullet}\mathrm{O}}_2\mathrm{H}+{\mathrm{H}}^{+}$$

15 $$\mathrm{CR}+\mathrm{C}{\mathrm{u}}^{+}\to \mathrm{carbon}\ \mathrm{radicals}+\mathrm{C}{\mathrm{u}}^{2+}$$

16 $$\mathrm{C}{\mathrm{u}}^{+}+{\mathrm{H}}_2{\mathrm{O}}_2\to \mathrm{C}{\mathrm{u}}^{2+}+{}{}^{\bullet}\mathrm{O}\mathrm{H}+\mathrm{O}{\mathrm{H}}^{-}$$

17 $$\mathrm{C}{\mathrm{u}}^{2+}+\mathrm{N}{\mathrm{i}}^{2+}\to \mathrm{C}{\mathrm{u}}^{+}+\mathrm{N}{\mathrm{i}}^{3+}$$

18 $$\mathrm{F}{\mathrm{e}}^{3+}+\mathrm{C}{\mathrm{u}}^{+}\to {\mathrm{Fe}}^{2+}+{\mathrm{Cu}}^{2+}$$

19 $${\mathrm{Co}}^{2+}+{\mathrm{H}}_2{\mathrm{O}}_2\to {\mathrm{Co}}^{3+}+{}{}^{\bullet}\mathrm{OH}+{\mathrm{O}\mathrm{H}}^{-}$$

20 $${\mathrm{Co}}^{3+}+{\mathrm{Fe}}^{2+}\to {\mathrm{Co}}^{2+}+{\mathrm{Fe}}^{3+}$$

21 $${\mathrm{Co}}^{3+}+{\mathrm{Cu}}^{+}\to {\mathrm{Cu}}^{2+}+{\mathrm{Co}}^{2+}$$

22 $${\mathrm{Mn}}^{2+}+{\mathrm{H}}_2{\mathrm{O}}_2\to {\mathrm{Mn}}^{3+}+{}{}^{\bullet}\mathrm{OH}+{\mathrm{O}\mathrm{H}}^{-}$$

23 $${\mathrm{H}}_2{\mathrm{O}}_2+{}{}^{\bullet}\mathrm{O}\mathrm{H}\to {\mathrm{H}}_2\mathrm{O}+{}{}^{\bullet }{\mathrm{O}}_2\mathrm{H}$$

24 $${\mathrm{M}}^{3+}+{{}{}^{\bullet}\mathrm{O}}_2\mathrm{H}\to {\mathrm{M}}^{2+}+{\mathrm{H}}^{+}+{\mathrm{O}}_2$$

25 $$\mathrm{CR}+{}{}^{\bullet}\mathrm{OH}\to \mathrm{by}\ \mathrm{products}\to {\mathrm{CO}}_2+{\mathrm{H}}_2\mathrm{O}$$

### The degradation pathway of CR

The formed intermediates during the CR degradation were determined via GC-MS analysis (Fig. S1), where the degradation of CR may occur through various steps: (a) cleavage of the C–S bond between the sulfonate groups and aromatic rings, (b) ring opening (cleavage of benzene ring), (c) breaking of azo bonds N=N, (d) cleavage of C–C and C–N bonds (Thomas et al. 2016). Nevertheless, it is impossible to identify all the intermediates of the degradation process as it is considered one of the GC-MS analysis limitations (Aghdasinia et al. 2016). Based on the GC-MS results, the CR degradation underwent asymmetric cleavage; initially, the two sulphonate groups of the CR dye were cleaved by ^•^OH, which was accompanied by withdrawing electrons from naphthalene rings, so ring opening was more favorable for naphthalene rings. The ring opening of benzene and naphthalene ring is done at ortho-position of ^•^OH groups resulting in obtaining diisooctyl phthalate (*RT*=23.27), which further degraded into dibutyl phthalate (*RT*=18.03). Then, the further oxidizing by ^•^OH generated benzene dicarboxylic acid (*RT*=27.66), n-hexadecanoic acid (*RT*=17.88), allyl phenol (*RT*=6.56), and phenylacetic acid (*RT*=5.02). Another pathway can be proposed through the asymmetric cleavage results in two molecules of sodium (4-amino-3-diazenyl naphthalene)-1-sulfonate and p-dihydroxy biphenyl. The latter compound can degrade to benzene propanoic acid (*RT*=24.90), which with further oxidation, produces phenol and 2-propenyl benzene (*RT*=6.30). In this investigation, it was noticed that asymmetric cleavage steps did not acquire any amino groups and overcome the production of naphthylamine and benzidine, which are tremendously toxic to the environment.

### The kinetic of H_2_O_2_ decomposition

**Fig. 10:**
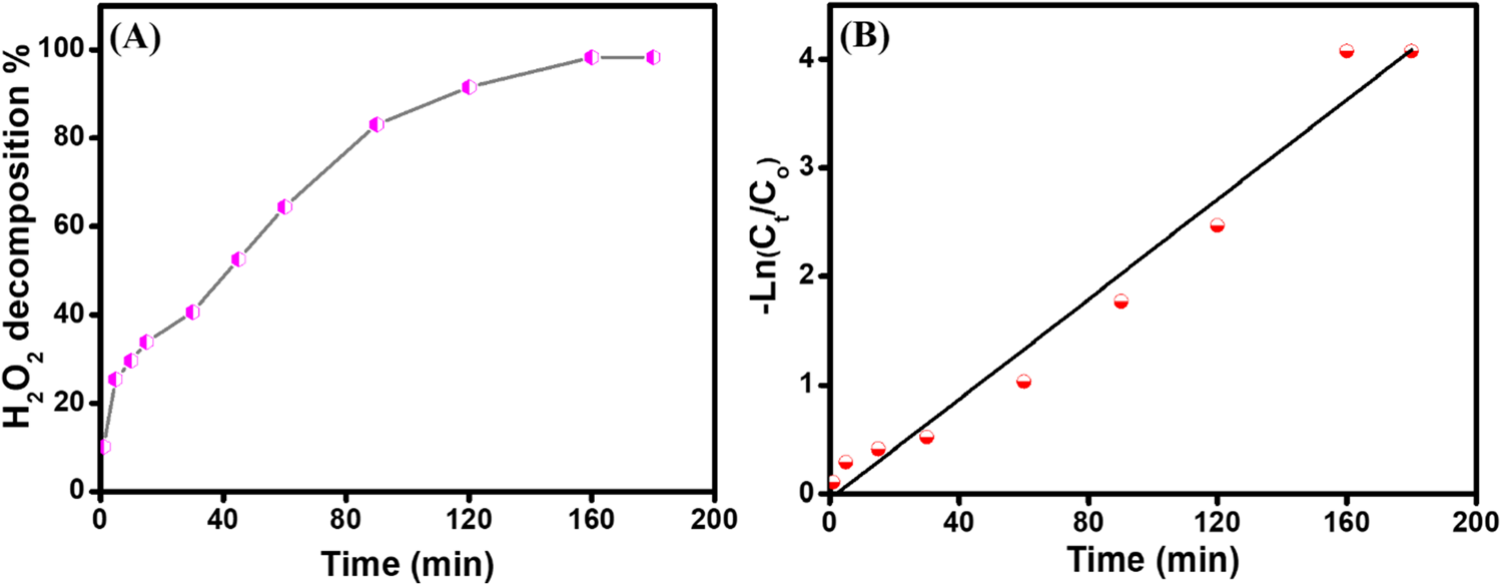
**A** Degradation of H_2_O_2_ with time and **B** first-order kinetic plot of H_2_O_2_ decomposition

Catalysts encompassing transition metals are potent catalysts for the decomposition of H_2_O_2_, where the catalyst donates electrons to accelerate the generation of radicals from H_2_O_2_ (Tatarchuk et al. 2020). Figure 10A elucidates the swift decomposition of H_2_O_2_ over Fe_3_O_4_@ZIF-67/CuNiMn-LDH catalyst in the absence of any organic pollutants. Before quantification via spectrophotometry, samples were withdrawn at each specified interval time and filtered. After 180 min, the decomposition efficiency of H_2_O_2_ reached 98.3%.

Figure 10B represented a plot between the decomposition degree and time, which indicates the decomposition reaction of H_2_O_2_ over Fe_3_O_4_@ZIF-67/CuNiMn-LDH catalyst fits first-order reaction kinetics. Therefore, the rate constant value was evaluated from the following equation.

26 $$\ln \left(\left[{\mathrm{H}}_2{\mathrm{O}}_2\right]\mathrm{t}/\left[{\mathrm{H}}_2{\mathrm{O}}_2\right]\mathrm{o}\right)=-{\mathrm{k}}_{\mathrm{obsd}}.\mathrm{t}$$

Where [H_2_O_2_]_t_ and [H_2_O_2_]_o_ are the hydrogen peroxides concentrations at time t and its initial concentration, respectively, and k_obsd_ is the first-order observed rate constant (Maharjan et al. 2020).

### Synergistic effect between adsorption and Fenton-like reaction

**Fig. 11:**
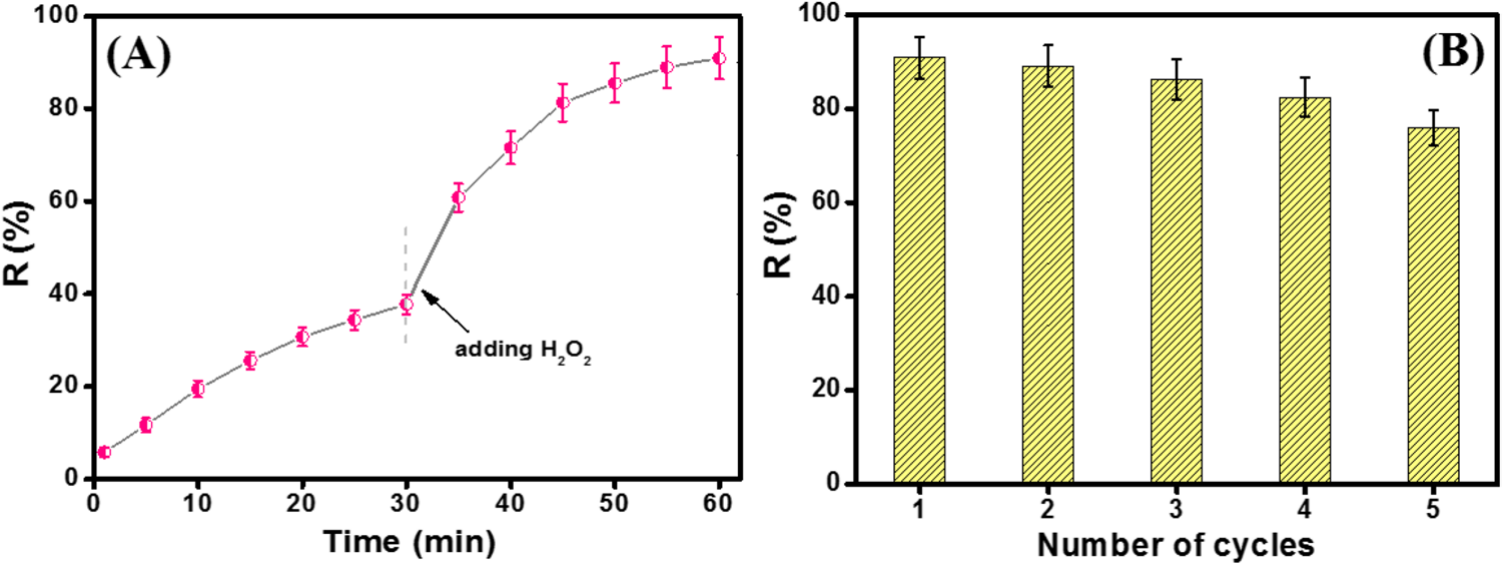
**A** Synergistic effect between adsorption and Fenton-like reaction in the degradation of CR, and **B** the recycle test of Fe_3_O_4_@ZIF-67/CuNiMn-LDH (*T*= 25 °C, *pH*=5, H_2_O_2_ concentration = 500 mg/L, catalyst dosage= 0.01 g, and CR concentration= 50 mg/L)

The adsorption role of Fe_3_O_4_@ZIF-67/CuNiMn-LDH in the degradation of CR was evaluated as follows: 10 mg of Fe_3_O_4_@ZIF-67/CuNiMn-LDH was added into 20 mL aqueous solution of CR with a concentration 50 mg/L at pH 5. A sample was withdrawn every 5 min, and measured its absorbance intensity via a UV-vis spectrometer. After 30 min, the adsorption of CR onto Fe_3_O_4_@ZIF-67/CuNiMn-LDH reached equilibrium, followed by adding 1 mL of H_2_O_2_ to initiate Fenton-like reaction. As shown in Fig. 11A, the adsorption % of CR onto Fe_3_O_4_@ZIF-67/CuNiMn-LDH slightly increased until attained its maximum percentage (37.77%) after 30 min. Interestingly, after starting Fenton-like reaction, the removal% of CR sharply increased to reach 90.9% within 30 min. This finding suggested the synergistic effect between adsorption and Fenton-like reaction to degrade CR molecules since the adsorbed CR around the active sites of the composite can be attacked effortlessly by the engendered ROS after the H_2_O_2_ addition (Lin et al. 2022).

### Recycle test

Figure 11B exhibits the degradation efficacy of the reused Fe_3_O_4_@ZIF-67/CuNiMn-LDH catalyst over five cycles, denoting the high stability of the catalyst since its catalytic activity towards the CR degradation is still high (75.9%). This diminution in the degradation efficacy can be assigned to the blocking of some active sites with the CR molecules during each cycle and also the possibility of losing some particles during the collection and washing stages.

### Metal leaching detection

**Fig. 12:**
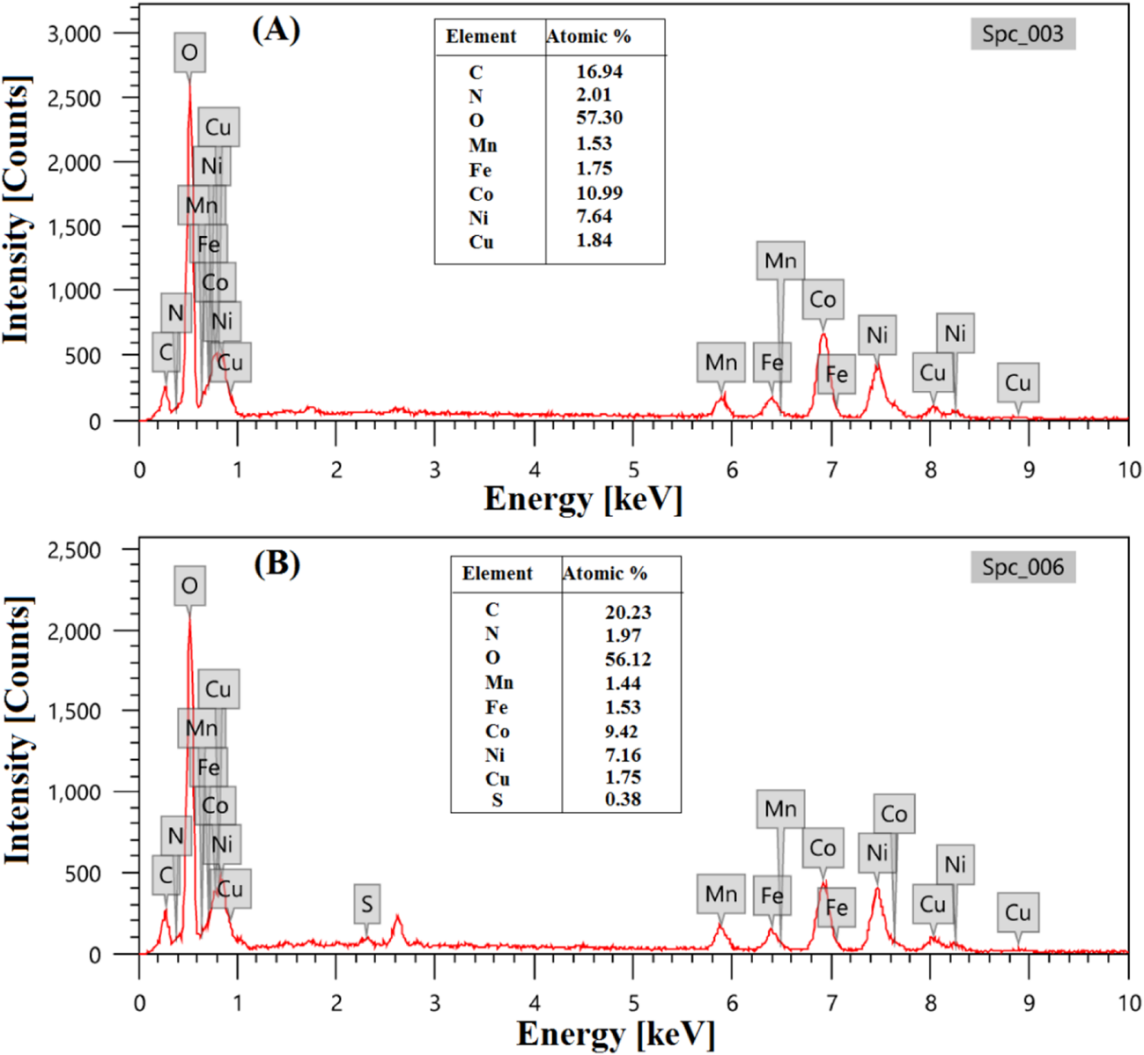
EDX patterns of Fe_3_O_4_@ZIF-67/CuNiMn-LDH; **A** before and **B** after the Fenton-like degradation of CR

For investigating the metal leaching from Fe_3_O_4_@ZIF-67/CuNiMn-LDH during the Fenton-like degradation of CR, the catalyst was analyzed before and after the degradation reaction by SEM-EDX. Figure 12A elucidates that Fe_3_O_4_@ZIF-67/CuNiMn-LDH consists of C, N, O, Mn, Fe, Co, Ni, and Cu with atomic % of 16.94, 2.01, 57.30, 1.53, 1.75, 10.99, 7.64, and 1.84%, respectively, while the EDX pattern of the catalyst after the Fenton-like degradation of CR (Fig. 12B) manifested the existence of S with an atomic % of about 0.38%. In addition, it was observed a slight metal leaching % of Mn, Fe, Co, Ni, and Cu of about 5.88, 12.57, 14.28, 6.28, and 4.89%, respectively.

Then, the concentrations of the leached metals were detected by ICP-MS to evince environmental friendliness, as well as assure the stability of Fe_3_O_4_@ZIF-67/CuNiMn-LDH. It was recorded that the leached concentrations of Mn, Fe, Co, Ni, and Cu were 0.083, 0.134, 0.159, 0.095, and 0.080 mg/L, which are lower than the legal limits. Notably, Fe_3_O_4_@ZIF-67/CuNiMn-LDH revealed higher durability compared to other relevant catalysts (Hou et al. 2019, Xu et al. 2016). This result confirmed the successful formation of continuous redox cycles between the contained transition metals in Fe_3_O_4_@ZIF-67/CuNiMn-LDH with extremely high ability to recover each other.

### Comparison study

Table 1 summarizes a comparative study between the degradation performance of Fe_3_O_4_@ZIF-67/CuNiMn-LDH and other reported catalysts in previous studies towards CR in different Fenton-like reaction conditions. Interestingly, Fe_3_O_4_@ZIF-67/CuNiMn-LDH exhibited superior catalytic activity toward CR (90.9%) within a shorter time and at room temperature. This propitious catalytic performance of our novel composite is most probably due to its unique morphology and synergistic effect between its components that form a continuous redox cycle. Although some catalysts revealed higher catalytic activity toward CR than Fe_3_O_4_@ZIF-67/CuNiMn-LDH, they required more complex degradation conditions and higher reaction time. For instance, the CeO_2_ nanorods catalyst reported by Wei et al. required higher H_2_O_2_ concentration and a long reaction time reached 2 h, in addition to Chen et al. deduced that the CR degradation by 4A-Fe@Cu catalyst took 12 h.

**Table 1:** Comparison between the catalytic degradation of Fe_3_O_4_@ZIF-67/CuNiMn-LDH and other catalysts toward CR

Catalysts	Parameters	CR removal %	Ref.
Fe_2_O_3_@CeO_2_–ZrO_2_/Pal	Time= 105 min, temperature= 30 °C; *pH*= 3.4; [H_2_O_2_] =40 mM; [CR] = 500 mg/L, catalyst= 4 g L^−1^	95%	(Ouyang et al. 2019)
MgFe_2_O_4_ nanorods	Time= 120 min, temperature= 80 °C, [CR]= 20 mg/L, catalyst = 40 mg, [H_2_O_2_] = 75 mg/L	95%	(Enlei et al. 2016)
FeCo@PAM	Time= 60 min, temperature= 35°C, [CR]= 100 mg/L, catalyst= 0.05 g, *pH*=7, H_2_O_2_= 1 mL	96.45%	(Aftab et al. 2019)
Ce-MOF	Time=180 min, [CR]=1000 mg/L, catalyst=10 mg, [H_2_O_2_] = (30 wt.%), 5 mL	80%	(Sharmoukh & Abdelhamid 2023)
Ni_0.6_Zn_0.4_Fe_2_O_4_	Time= 60 min, [CR]=20 mg L^−1^, catalyst= 1 g/L, *pH*=7.4, v(H_2_O_2_)/v(H_2_O)=20	92.07%	(Nadjia et al. 2017)
CeO_2_ nanorods	Time= 120 min, temperature= 25 °C, [CR]= 70 mgL^−1^, catalyst= 1 g/L, [H_2_O_2_] = 20 mM, *pH*=9	98%	(Wei et al. 2019)
Mn_2_O_3_−Co_3_O_4_/C	Time=15 min, temperature= 45 °C, [CR]=10 mg L^−1^, catalyst= 3 mg, H_2_O_2_= 1 mL	88.4%	(Hazarika et al. 2020)
4A-Fe@Cu	Time=12 h, [CR]= 1 g L^−1^, catalyst=2 g/L, H_2_O_2_= 7.2 mM, *pH*=8	99.2%	(Chen et al. 2022)
Fe_3_O_4_@ZIF-67/CuNiMn-LDH	Time= 30 min, temperature= 25 °C [CR] = 50 mg/L, catalyst = 0.01 g, [H_2_O_2_] = 500 mg/L, *pH*= 5	90.9%	This study

## Conclusions

In summary, a novel CuNiMn-LDH was synthesized and modified by Fe_3_O_4_ and ZIF-67, forming a remarkable heterogeneous Fenton-like Fe_3_O_4_@ZIF-67/CuNiMn-LDH catalyst that revealed a synergistic effect between catalytic activity of the components for superb degradation of CR. The acquired results from FTIR, XRD, XPS, SEM-EDX, and SEM studies emphasized the successful preparation of Fe_3_O_4_@ZIF-67/CuNiMn-LDH. Furthermore, VSM revealed the soft ferromagnetic nature of Fe_3_O_4_@ZIF-67/CuNiMn-LDH, and ZP defined that its pH_zpc_ was ~5.8. Significantly, the CR degradation accomplished 90.9% by the Fe_3_O_4_@ZIF-67/Cu Ni Mn-LDH/H_2_O_2_ system within 30 min with optimum conditions (*pH*= 5, catalyst dosage= 0.01, H_2_O_2_ concentration= 500 mg/L, temperature= 25 °C, and CR concentration = 50 mg/L). Owing to the synergistic effect between Fe, Cu, Co, Ni, and Mn, Fe_3_O_4_@ZIF-67/CuNiMn-LDH catalyst exhibited excellent catalytic activity, in addition to its superb catalytic activity towards other cationic and anionic dyes. Interestingly, it was concluded from the quenching test that the CR degradation by the Fe_3_O_4_@ZIF-67/Cu Ni Mn-LDH/H_2_O_2_ system proceeded via the radical pathway. This finding was evinced by the mechanism study based on XPS analysis that confirmed the formation of a continuous redox cycle, generating abundant electrons for the H_2_O_2_ activation and the ^•^OH production.

## Supplementary information

ESM 1 The online version contains supplementary material available at XXX. (DOCX 120 kb)

## Data Availability

The datasets used and analyzed during the current study are available from the corresponding author on reasonable request.
