# Supplementary material for: Magnetic hierarchical flower-like Fe3O4@ZIF-67/CuNiMn-LDH catalyst with enhanced redox cycle for Fenton-like degradation of Congo red: optimization and mechanism
Source: Environ Sci Pollut Res Int. 2023 May 23;30(30):75332–48. doi: 10.1007/s11356-023-27430-2 (PMC10293427; doi:10.1007/s11356-023-27430-2)

# **Magnetic hierarchical flower-like Fe_3_O_4_@ZIF-67/CuNiMn-LDH catalyst with enhanced redox cycle for Fenton-like degradation of Congo red: Optimization and mechanism**

Abdelazeem S. Eltaweil, Sara S. Bakr, Eman M. Abd El-Monaem^*^, Gehan M. El-Subruiti

Chemistry Department, Faculty of Science, Alexandria University, Alexandria, Egypt.

Corresponding authors email: Eman M. Abd El-Monaem (emanabdelmonaem5925@yahoo.com).

**Supplementary Information**

**Text S1**

Ferric chloride hexahydrate (FeCl_3_.6H_2_O), iron sulfate heptahydrate (FeSO_4_.7H_2_O), and ammonium hydroxide (99%) were purchased from Aladdin Industrial Corporation (China). Copper chloride dihydrate (CuCl_2_.2H_2_O), nickel chloride dihydrate (NiCl_2_.2H_2_O), Ethanol (C_2_H_5_OH; 99.9%), and 2-methylimidazole (MeIm) was obtained from Sigma-Aldrich (Germany). N,N-dimethylformamide (DMF, ≥ 99.8 %), hydrogen peroxide (H_2_O_2_ ; 35%), and hydrochloric acid (37%) were provided from Rankem (India). Sodium hydroxide (NaOH, 99.5%), manganese chloride tetrahydrate (MnCl_2_.4H_2_O, 99.99%), and cobalt nitrate hexahydrate (Co(NO_3_)_2_⋅6H_2_O) were supplied from Alpha Chemika (India). Congo red, crystal violet (CV), Methyl orange (MO), and methylene blue (MB) were obtained from MP Biomedicals LLC (France).

**Table S1:** the derived parameters of pseudo-first-order kinetic

| **CR concentration (mg/L)** | **k** | **R^2^** |
| --- | --- | --- |
| **50** | 0.0341 | 0.9449 |
| **100** | 0.012 | 0.8963 |
| **200** | 0.0089 | 0.9723 |
| **300** | 0.0072 | 0.9734 |

Fig. S1 GC-MS spectra of CR (A) before, and (B) after the Fenton-like degradation reaction


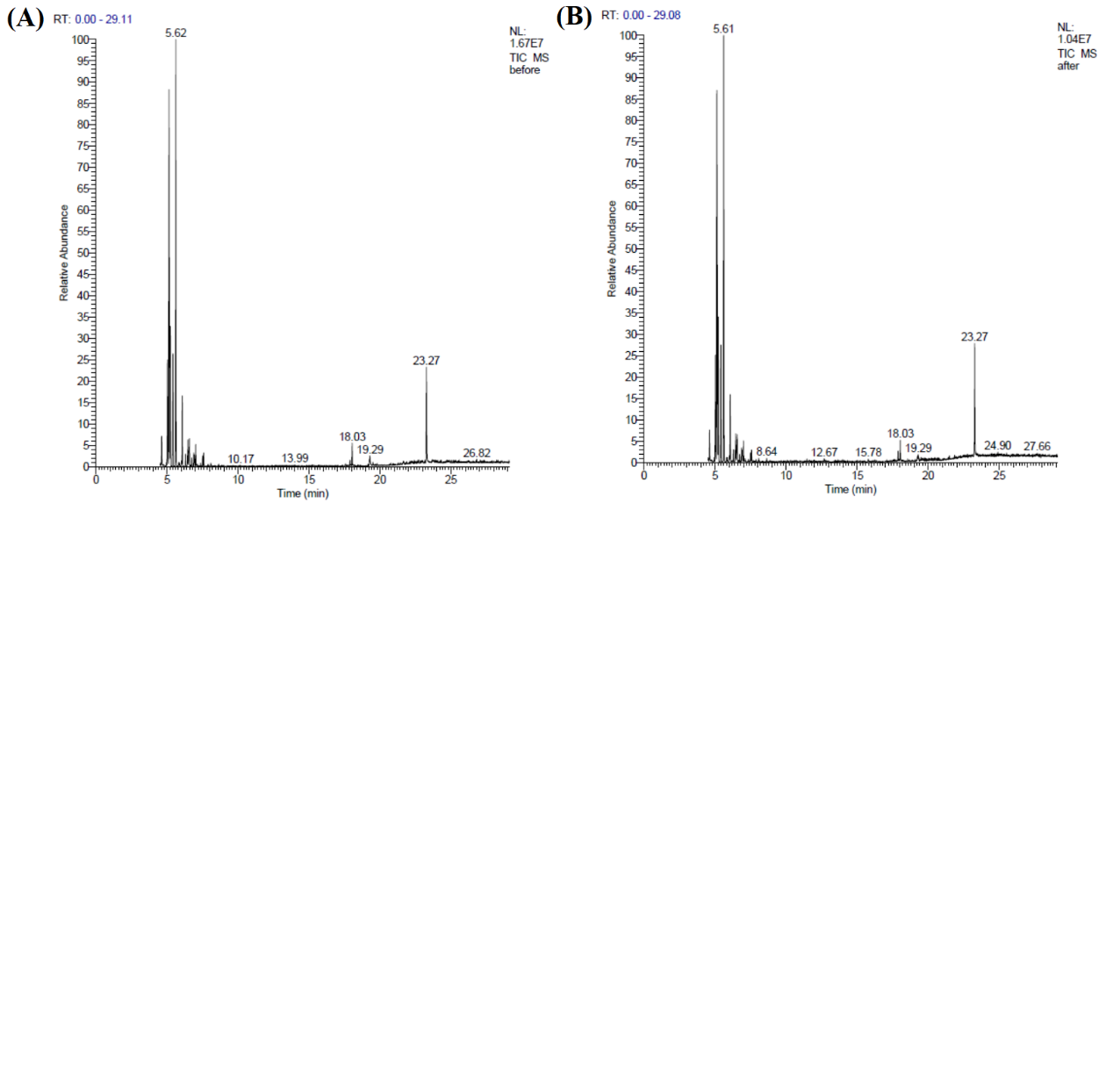

Supplement: Supplementary file 1 — The online version contains supplementary material available at XXX. (DOCX 120 kb) [file 11356_2023_27430_MOESM1_ESM.docx]
